# *In silico* identification and characterization of *AGO*, *DCL* and *RDR* gene families and their associated regulatory elements in sweet orange (*Citrus sinensis* L.)

**DOI:** 10.1371/journal.pone.0228233

**Published:** 2020-12-21

**Authors:** Md. Parvez Mosharaf, Hafizur Rahman, Md. Asif Ahsan, Zobaer Akond, Fee Faysal Ahmed, Md. Mazharul Islam, Mohammad Ali Moni, Md. Nurul Haque Mollah

**Affiliations:** 1 Bioinformatics Laboratory, Department of Statistics, University of Rajshahi, Rajshahi, Bangladesh; 2 Department of Microbiology, Rajshahi Institute of Biosciences, University of Rajshahi, Rajshahi, Bangladesh; 3 Institute of Environmental Science, University of Rajshahi, Rajshahi, Bangladesh; 4 Agricultural Statistics and ICT Division, Bangladesh Agricultural Research Institute (BARI), Gazipur, Bangladesh; 5 Department of Mathematics, Jashore University of Science and Technology, Jashore, Bangladesh; 6 The University of Sydney, Sydney Medical School, School of Medical Sciences, Discipline of Biomedical Science, Sydney, New South Wales, Australia; Institute for Biological Research "S. Stanković", University of Belgrade, SERBIA

## Abstract

RNA interference (RNAi) plays key roles in post-transcriptional and chromatin modification levels as well as regulates various eukaryotic gene expressions which are involved in stress responses, development and maintenance of genome integrity during developmental stages. The whole mechanism of RNAi pathway is directly involved with the gene-silencing process by the interaction of Dicer-Like (*DCL*), Argonaute (*AGO*) and RNA-dependent RNA polymerase (*RDR*) gene families and their regulatory elements. However, these RNAi gene families and their sub-cellular locations, functional pathways and regulatory components were not extensively investigated in the case of economically and nutritionally important fruit plant sweet orange (*Citrus sinensis* L.). Therefore, *in silico* characterization, gene diversity and regulatory factor analysis of RNA silencing genes in *C*. *sinensis* were conducted by using the integrated bioinformatics approaches. Genome-wide comparison analysis based on phylogenetic tree approach detected 4 *CsDCL*, 8 *CsAGO* and 4 *CsRDR* as RNAi candidate genes in *C*. *sinensis* corresponding to the RNAi genes of model plant *Arabidopsis thaliana*. The domain and motif composition and gene structure analyses for all three gene families exhibited almost homogeneity within the same group members. The Gene Ontology enrichment analysis clearly indicated that the predicted genes have direct involvement into the gene-silencing and other important pathways. The key regulatory transcription factors (TFs) MYB, Dof, ERF, NAC, MIKC_MADS, WRKY and bZIP were identified by their interaction network analysis with the predicted genes. The *cis*-acting regulatory elements associated with the predicted genes were detected as responsive to light, stress and hormone functions. Furthermore, the expressed sequence tag (EST) analysis showed that these RNAi candidate genes were highly expressed in fruit and leaves indicating their organ specific functions. Our genome-wide comparison and integrated bioinformatics analyses provided some necessary information about sweet orange RNA silencing components that would pave a ground for further investigation of functional mechanism of the predicted genes and their regulatory factors.

## Introduction

In multicellular eukaryotes, wide range of biological functions including genome rearrangement, antiviral defense, heterochromatin formation and development patterning and timing are fine-tuned by generally two types of small RNA (sRNA; including 21–24 nucleotides), named microRNA (miRNA) and short interfering RNA (siRNA) [1–3]. These sRNA molecules are involved in both transcriptional and post-transcriptional gene silencing as well as natural immunity system [2, 4–7]. In plants, the sRNA biogenesis process is significantly regulated by the proteins encoded by respective Dicer-like (*DCL*), Argonate (*AGO*) and RNA-dependent RNA polymerases (*RDR*) gene families. In plants, RDRs are inevitable gene silencing members that synthesize dsRNA by using RNA template and actually intensify the gene silencing signals [8–13]. The DCLs are responsible for the cleavage of dsRNAs into 21–24 nucleotide long small RNAs (i.e. siRNA or miRNA). The specification to the endonuclease-containing, RNA-induced silencing complex (RISC) is provided by these sRNAs which facilitate the AGO proteins with RNaseH-type activities to degrade the target homologous RNAs with the sequence complementary to the small RNAs [14, 15]. These are also involved in the transcriptional gene silencing by the implementation of chromatin reformation [16, 17].

DCL proteins, which mainly process the small mature RNAs from the long double-stranded RNAs [18–22] are a major component of RNA interference (RNAi) pathway (also known as small RNA (sRNA) biogenesis process). The DCL proteins have the functional domains, named DEAD/ResIII, Helicase_C, Dicer_Dimer, PAZ, RNase III and DSRM [23] which play an important role for the proteins to be functional. The PAZ domain acts to bind the siRNA as well as the dsRNA which is cleaved by the two catalytic RNaseIII domains. The main components of RNAi are the AGO proteins which play the core role of gene silencing [24]. All the AGO proteins include the Argo-N/Argo-L, PAZ, MID and PIWI significant functional domains [14]. A significant specific binding pocket is contained in the PAZ domain. Additionally, to anchor the sRNA onto the AGO proteins, the specific pocket of MID domain binds the 5*'* phosphate of the small RNAs [25]. The siRNA 5*'* end is bonded to the target RNA by the PIWI domain [26]. Among the three groups of AGO proteins i.e. Ago -like, PIWI-like and *C*. *elegens-*specific group 3 AGO proteins [27, 28], the AGO-like proteins are presented and expressed in plants, animals, fungi and bacteria, while PIWI-like proteins have only been found in animals [29]. Some important catalytic residues are missed by *C*. *elegens -*specific group 3 AGO proteins [27] while the other AGOs conserved them and the expression of PIWI-like group is restricted in human germ-cell and in rat and some mammals [29]. The third major RNAi associated protein is RDR which has not been identified in insects or vertebrates [30] but is present in fungi, nematodes and plants. The only special conserved catalytic RNA-dependent RNA polymerase (RdRP) domain is shared by the RDR which makes the RDR proteins a consistent member of RNAi gene family [31–33]. Among the three types of RDR: RDRα, RDRβ and RDRγ, the RDRβ group has not been found in plants [31, 32].

In case of plants, the *DCL*, *AGO* and *RDR* gene families related to distinct RNAi pathways [34–36] vary from 20 genes in *Arabidopsis* [37] to 51 genes in *Brassica* [38] species. The member of these RNAi associated gene families has been identified in many plants species such as 32 genes in rice [14], 28 genes in maize [39] and tomato [33], 38 genes in foxtail millet [40], 22 genes in grapevine [41] and pepper [42] and 20 genes in cucumber [43]. Recently 23 genes in Barley (*Hordeum vulgare L*.) [44], 36 genes in sugarcane (*Saccharum spontaneum*) [45] and 25 genes in sweet orange (*Citrus sinensis)* [46] belonging to *DCL*, *AGO* and *RDR* genes have been identified and characterized. Besides, their expression pattern was also investigated under various conditions.

In *A*. *thaliana*, AtDCL1, AtDCL3 and AtAGO4 influenced the RNA-directed DNA methylation of the *FWA* transgene linkage to the histone H3 lysine 9 (H3K9) methylation [47, 48]. AtDCL2 is associated with the virus defence and siRNA production while the AtDCL4 is related to the regulation of vegetative phase change [23, 49]. AtDCL1 and AtDCL3 function for *A*. *thaliana* flowering [50]. Moreover, in rice if the OsDCL1 is knocked down then it fails to perform siRNA metabolism which causes pleiotropic phenotype in rice [51]. Besides, AGO proteins related to various forms of RNA silencing, such as AtAGO1 is associated with the transgene-silencing pathways [52] and AtAGO4 with the epigenetic silencing [47]. AtAGO7 and AtAGO10 influence the plant growth [53] and meristem maintenance [54]. Additionally, other AGOs also have a significant role in RNAi pathways. On the other hand, previous studies reported that the RDR genes are responsible for different gene silencing including co-suppression, virus defence, chromatin silencing and PTGS in plants such as in *A*. *thaliana* and maize [11, 35, 55–57]. Also the RDRα type enzyme was recognized playing a vital role in endogenous gene regulation [15, 58] antiviral silencing [8, 59, 60], arrangement of heterochromatin and genome resistance [61, 62].

The sweet orange (*C*. *sinensis*) is considered a great natural source of vitamin C, antioxidants and high nutrition important for the human body [46, 63–65]. It is considered the second highest amount of fruit producing plant all over the world (FAO Statistics 2006) and around USD 9 billion estimated price value was reported for the total production of sweet orange in 2012 [46, 66]. It not only has the market value, but also contains about 170 phytonutrients and over 60 flavonoids which work as antioxidant, anti-inflammation, anti-cancer and anti-arteriosclerosis compounds. It also protects us from many chronic diseases like arthritis, obesity and coronary heart diseases [67–70]. In spite of extensive studies of RNAi-related genes in many other plant species, very little information is available in the literature about these gene families for sweet orange. Until now, 13 *AGO*, 5 *DCL* and 7 *RDR* genes have been identified and investigated regarding their roles in fruit abscission process in *C*. *sinensis* [46]. However, sub-cellular location, functional pathways and associated regulatory factors (transcription and *cis*-acting) of these gene families in *C*. *sinensis* are not yet widely investigated. Therefore, in this study, an attempt is made to accomplish a comprehensive *in silico* analyses for genome-wide identification and characterization of *AGO*, *DCL* and *RDR* gene families and their associated regulatory elements in *C*. *sinensis*. Our results provide first insights into the genome-wide composition study, predicted function and factors influencing regulatory process of RNAi pathway genes in *C*. *sinensis*.

## Materials and methods

### Data source of *DCL*, *AGO* and *RDR* genes

**Fig 1:**
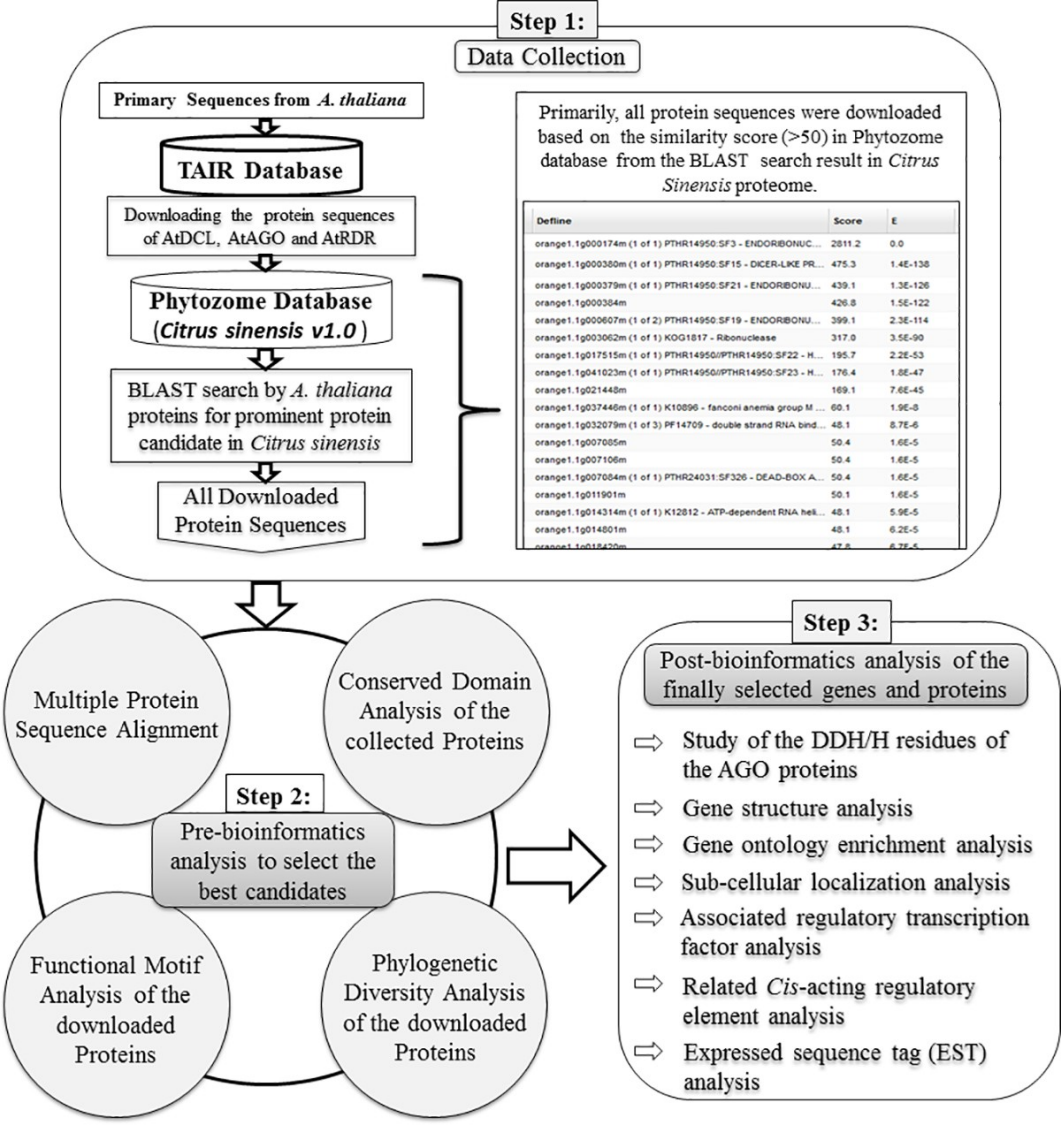
The working flowchart of the integrated bioinformatics analyses approaches to select the best candidates for *DCL*, *AGO* and *RDR* genes and their associated regulatory elements in *C*. *sinensis*.

For genome-wide identification of *DCL*, *AGO* and *RDR* genes in sweet orange (*C*. *sinensis*), protein sequences were downloaded from the Phytozome database (https://phytozome.jgi.doe.gov/pz/portal.html) by taking advantage of completed *C*. *sinensis* genome sequence [64]. The previously identified sRNA biogenesis protein sequences of the model plant *A*. *thaliana* (AtDCLs, AtAGOs and AtRDRs) were collected from The Arabidopsis Information Resource (TAIR) (http://www.arabidopsis.org) and used to search the protein sequence of *C*. *sinensis*. The Basic Local Alignment Search Tool (BLASTP) program was used against *C*. *sinensis* genome in the Phytozome database (Fig 1).

The derived paralog protein sequences from *C*. *sinensis* were downloaded with the significant score (≥50) and the significant *E*-values. For avoiding the redundancy of sequences, only the primary transcripts were considered in this analysis. The conserved domains of all retrieved sequences were searched and predicted by using the Pfam (http://pfam.sanger.ac.uk/) and the NCBI-CD database (http://www.ncbi.nlm.nih.gov/Structure/cdd/wrpsb.cgi) and the SMART analysis. By this time, the different genomic information including the primary transcript name, genomic length and the chromosomal location of genes, ORF length, encoded protein length was downloaded from the *C*. *sinensis* genome in Phytozome database. In this study, the computationally identified new *CsDCLs*, *CsAGOs* and *CsRDRs* genes in *C*. *sinensis* genome were named according to the nomenclature based on phylogenetic relatedness of the similar family-members of the *A*. *thaliana* genes as named previously. The molecular weight of the selected protein sequences was predicted by using the ExPASyComputepI/Mwtool (http://au.expasy.org/tools/pitool.html).

### Integrated bioinformatics analyses approaches

The integrated bioinformatics analyses approaches which included the sequence alignment and phylogenetic tree construction, prediction of the functional domain and motif structure of the proteins, the exon-intron structure of the RNAi candidate genes, gene ontology (GO) analysis, prediction of subcellular location, regulatory network among the gene transcription factors and *C*. *sinensis* RNAi candidate genes, *cis*-acting regulatory element (CARE) analysis, express sequence tag (EST) analysis, were carried out for comprehensive genome-wide identification, characterization, diversification analysis and to retrieve regulatory transcription components of *C*. *sinensis* RNA silencing machinery genes (Fig 1). These approaches are described in the following sub-sections.

### Sequence alignment and phylogenetic analysis

In this *in silico* identification, the multiple sequence alignments of the encoded protein sequences of the predicted genes were conducted by using the Clustal-W method [71] with the MEGA5 program [72]. Finally, the phylogenetic tree analysis was carried out using the Neighbor-joining method [73] implemented on the aligned sequenced and the 1,000 bootstrap-replicates [74] were used to check this evolutionary relationship. The evolutionary distances were computed using the Equal Input method [75].

### Conserved domain and motif analysis

**Fig 2:**
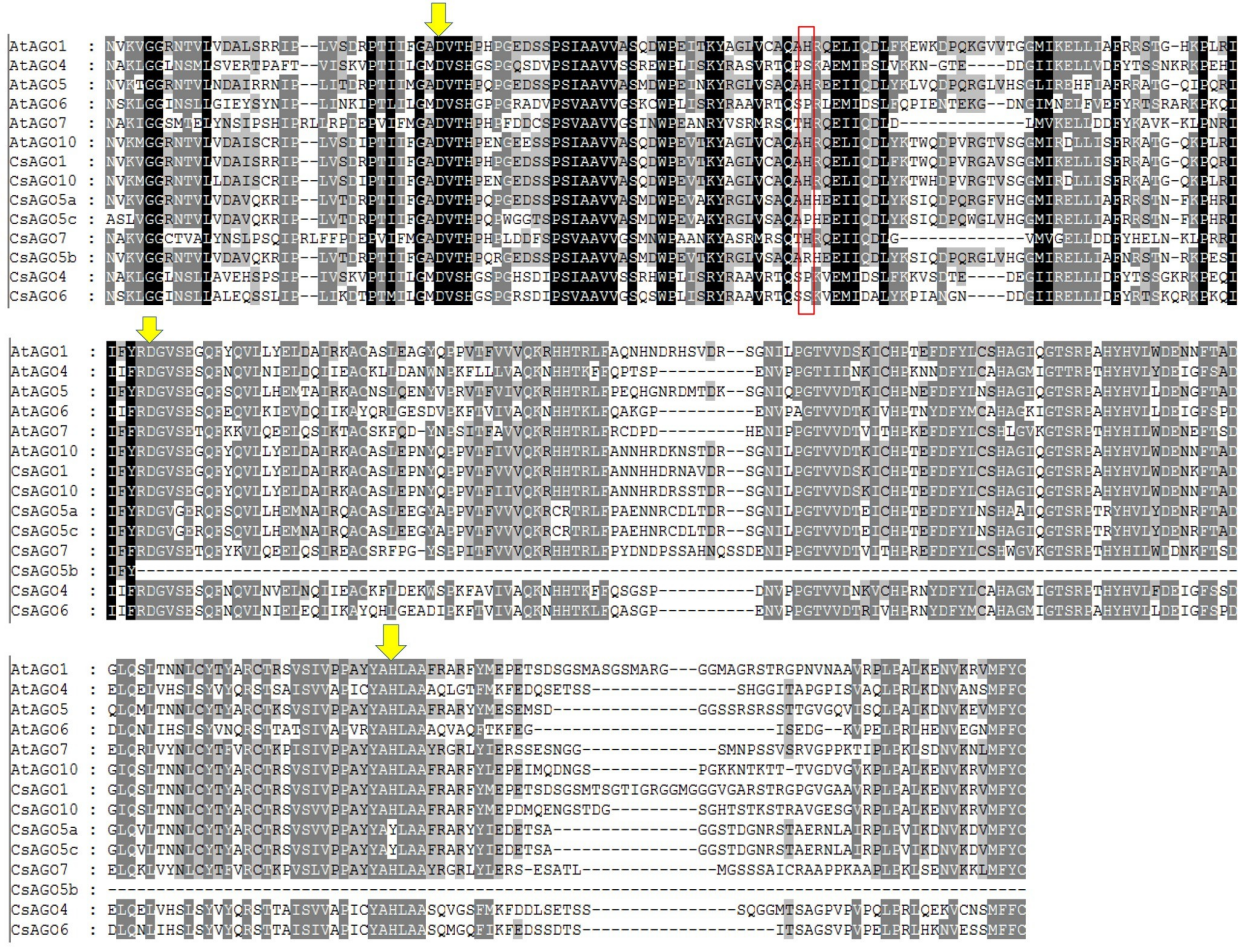
The multiple sequence alignment profile of PIWI domain of the amino acids sequences of *C*. *sinensis* and *Arabidopsis* AGO proteins by Clustal-W program in MEGA5. The downward yellow arrows indicate the position of conserved DDH triad of PIWI domain and the conserved H798 positions are surrounded by red box.

To investigate the functional domains of the predicted genes the NCBI-CDD, Pfam database and SMART analysis were utilized to retrieve the conserved domains. The reported RNAi related proteins in *C*. *sinensis* containing the maximum number of significant functional domains similar to the *Arabidopsis* proteins AtDCLs, AtAGOs and AtRDRs were selected. In motif investigation, the most significant conserved metal-chelating catalytic triad residues in the PIWI domain of AGO proteins, i.e. aspartate, aspartate and histidine (DDH) [14] as well as histidine at 798 positions (H798) were investigated for reported CsAGO proteins (Fig 2). The conserved motif divergences among all the predicted RNAi related proteins were conducted by a complete online program for protein sequence analysis i.e. Multiple Expectation Maximization for Motif Elicitation (MEME-Suite) [76]. For this purpose, the following parameters were specified: (i) optimum motif width as ≥6 and ≤50; (ii) maximum 20 motifs.

### Gene structure and genomic location analysis

The gene structure of the predicted genes was constructed using the online Gene Structure Display Server (GSDS 2.0, http://gsds.cbi.pku.edu.cn/index.php) [77]. The structures of the selected genes were compared with the gene structure of *A*. *thaliana* to compare the exon-intron composition of the predicted genes in *C*. *sinensis*. The genomic location of the reported genes were represented using online tool MapGene2Chromosome V2 (http://mg2c.iask.in/mg2c_v2.0/).

### Gene ontology and sub-cellular localization analysis

To check the engagement of our predicted RNAi associated genes with the cluster of different biological processes and molecular functional pathways, the Gene Ontology (GO) analysis was conducted using online tool implemented in PlantTFDB [78]. Here, the respective *p*-values were determined by Fisher’s exact test and Benjamini-Hochberg’s corrections. We considered the *p*-value < 0.05 as statistically significant for the GO enrichment results corresponding to the predicted genes. For the reported gene products, the sub-cellular location was investigated into the cell considering the different organelles. Web-based integrative subcellular location predictor tool called plant subcellular localization integrative predictor (PSI) [79] was used to predict the subcellular location of the identified genes.

### Regulatory relationship and network analyses between TFs and *C*. *sinensis* RNAi related genes

In this study, the analysis of associated TFs family with the predicted RNAi related genes in *C*. *sinensis* was conducted from the widely used plant transcription factor database, PlantTFDB (http://planttfdb.cbi.pku.edu.cn//). After identification of the related regulatory TFs of the *C*. *sinensis* RNAi associated genes, the regulatory network and sub-network were constructed and visualized using Cytoscape 3.7.1 [80] to find out the hub proteins and the related important hub TF through the interaction network. The key hub factors were selected based on the highest degree of connectivity into the interaction network. The networks were constructed to investigate the key regulatory relationship between the TFs and reported RNAi related genes.

### *Cis*-regulatory element analysis

To investigate *cis*-elements in the promoter sequences of three RNAi-related (*CsDCL*, *CsAGO* and *CsRDR*) gene families, 1.5 kb sequences upstream of the initiation codon (ATG) were collected and subjected to stress response-related *cis*-acting element online prediction analysis with Signal Scan search program in the PlantCARE database (http://bioinformatics.psb.ugent.be/webtools/plantcare/html/) [81]. The collected *cis-*regulatory element was classified into five categories: light responsive (LR), stress responsive (SR), hormone responsive (HR), other activities (OT) and unknown function. The known and established *cis*-elements of *CsDCLs*, *CsAGOs* and *CsRDR* are represented separately.

### *In silico* Expressed Sequence Tag (EST) analysis

For the important and valuable information about the gene expression, the *in silico* expressed sequence tag (EST) data analysis was conducted according to Mirzaei et al., 2014 [82] for the reported genes. The PlantGDB database (http://www.plantgdb.org/cgi-bin/blast/PlantGDB/) was used for EST mining against the proposed RNAi related genes in *C*. *sinensis*. The default parameter with *e*-value = 1e-10 was considered for BLASTN search for the EST mining in PlantGDB database. The PlantGDB is a regularly updated online platform where the EST data from NCBI-dbEST and GeneBank are accessible [83]. The further heatmap was constructed to represent the specific RNAi associated gene expression into different tissue and organ in this fruit plant.

## Results and discussion

### Identification and characterization of *CsDCLs*, *CsAGOs* and *CsRDRs* genes

To identify the best candidates of RNAi related pathway in *C*. *sinensis* similar to the *A*. *thaliana*, all the previously downloaded sequences were gone through various kinds of analysis (Fig 1). Finally, we have identified 4 *DCL*, 8 *AGO* and 4 *RDR* genes encoding CsDCLs, CsAGOs and CsRDRs proteins, respectively, in the *C*. *sinensis* genome. On the basis of HMMER analysis with regards of all six types of conserved domains DEAD, Helicase_C, Dicer_dimar, PAZ, RNase III and DSRM; four DCL loci were identified in sweet orange genome. The genome length of predicted *CsDCL* genes varied from 10603 bp to 12728 bp corresponding to *CsDCL1* and *CsDCL2* with the coding potentiality of 1931 and 1396 amino acids (Table 1) when the ORF varied from 4191 bp (*CsDCL2*, orange1.1g000607m) to 5796 bp (*CsDCL1*, orange1.1g000174m). This findings are similar with Sabbione et al., 2019 except for the CsDCL2b (orange1.1g003062) protein which was additionally reported [46]. In this analysis the identified *CsDCLs* genes did not have any paralogs within the four subgroups in *C*. *sinensis*. The isoelectric point (pI) values of the CsDCLs proteins indicated that the proteins are more likely to be acidic where only the CsDCL3 have the highest pI value 8.01.

**Table 1:** Basic information about *C*. *sinensis* Dicer-like, Argonaute and RNA-dependent RNA polymerase gene families.

Serial Number	Gene Name	Accession Number	Chromosomal location	ORF length (bp)	Gene Length (bp)	Protein			
						No. of Intron	Molecular Weight (Da)	Protein Length (aa)	pI
*CsDCLs*									
1	CsDCL1	orange1.1g000174m	scaffold00001:3480331..3490933	5796	10603	18	216424.20	1931	5.96
2	CsDCL2	orange1.1g000607m	scaffold00367:74912..87639	4191	12728	21	158487.91	1396	7.65
3	CsDCL3	orange1.1g000379m	scaffold00013:998934..1009643	4815	10710	24	178949.30	1604	8.01
4	CsDCL4	orange1.1g000380m	scaffold00068:219006..230745	4806	11740	25	179592.47	1601	6.46
*CsAGOs*									
1	CsAGO1	orange1.1g001466m	scaffold00674:51962..59926	3222	7965	20	118338.09	1073	9.38
2	CsAGO4	orange1.1g002449m	scaffold00028:907375..916121	2763	8747	21	102997.53	920	8.98
3	CsAGO5a	orange1.1g002204m	scaffold00595:85931..92382	2865	6452	21	106772.65	954	9.27
4	CsAGO5b	orange1.1g037086m	scaffold00595:96826..99593	1278	2768	10	48003.28	426	9.05
5	CsAGO5c	orange1.1g003630m	scaffold03700:12..6517	2421	6506	21	91163.31	806	9.03
6	CsAGO6	orange1.1g002661m	scaffold00067:338566..346634	2688	8069	21	100499.54	895	9.41
7	CsAGO7	orange1.1g001684m	scaffold00003:3309646..3313553	3093	3908	2	117035.78	1030	9.24
8	CsAGO10	orange1.1g001954m	scaffold00011:54251..63917	2979	9667	20	111515.83	992	9.34
*CsRDRs*									
1	CsRDR1	orange1.1g002586m	scaffold00058:231382..236081	2715	4700	2	103382.03	904	6.51
2	CsRDR2	orange1.1g001183m	scaffold00020:1255446..1250927	3396	4520	3	128958.15	1131	6.59
3	CsRDR3	orange1.1g001771m	scaffold00027:638984..650509	3048	11526	18	115670.83	1015	6.80
4	CsRDR6	orange1.1g041430m	scaffold d00051:398116..402488	3594	4373	1	136437.20	1197	6.13

[Information containing the gene names, accession number, chromosomal location, ORF length, genome length, protein length were collected from Phytozome database (https://phytozome.jgi.doe.gov/pz/portal.html) and the molecular weight and isoelectric point (pI) values were predicted by the ExPASy ComputepI/Mwtool (http://au.expasy.org/tools/pi_tool.html). Molecular weights are in Da (Daltons) and “aa” means amino acid.]

Based on the conserved domain PAZ and PIWI from the putative polypeptide sequences by HMM and HMMER analysis, we have isolated a total of 8 *AGO* genes in the *C*. *sinensis* genome. Conserved domain analysis by the Pfam database, NCBI databases and SMART analysis reported that all the selected AGO proteins (CsAGO1-8) shared an N-terminus PAZ domain and a C-terminus PIWI super family domain that are the core properties of plant AGO proteins.

From the previous study, it is observed that the PIWI domain demonstrating expansive homology to RNase H binds the siRNA 5*'* end to the target RNA [26] and cracks the target RNAs that represent the complementary sequences to small RNAs [84]. Interestingly, the catalytic trait, three conserved metal-chelating residues (D = aspartate, D = aspartate and H = histidine) in PIWI domain, are related to the previous event and this trait was firstly shown in the model plant *A*. *thaliana* on AGO1 [14].

Moreover, another critical conserved histidine residue in AGO1 for *in vitro* endonuclease activity [85] was found. The genome length of the selected *CsAGO* genes varied from 2768 bp to 9667 bp produced by the *CsAGO5b* (orange1.1g037086m) and *CsAGO10* (orange1.1g001954m), respectively, with the coding potentiality of 426 and 992 amino acids. The genes having the ORF ranging from 1278 to 3222 bp (CsAGO5b and CsAGO1) encode the reported CsAGO proteins homologous. In this study, the multiple sequence alignment of the PIWI domains of all CsAGO proteins with the orthologs AtAGOs from *A*. *thaliana* using the CLUSTAL-W method was utilized (Fig 2).

This alignment revealed that the five CsAGO proteins represented the conserved DDH triad residues like *A*. *thaliana* AGO1. We also investigated the important DDH/H motif among the reported CsAGO proteins. The DDH/H motif was found in CsAGO1, CsAGO7 and CsAGO10 proteins where the DDH/P motif and the DDH/S motif were identified in the CsAGO4 and CsAGO6 protein. The DDY/H motif and the DDY/P motif were found in CsAGO5a and CsAGO5c protein, respectively.

Among the CsAGO proteins, the CsAGO5b represented two missing PIWI domain catalytic residue(s) in the second aspartate at the 845^th^ position (D845) and third histidine at the 986^th^ position (H986) (Table 2). But the other two CsAGO proteins, CsAGO5a and CsAGO5c had the catalytic trait but with a replacement in the third histidine residue at the 986^th^ position by tyrosine (Y) residue. Therefore, the DDH catalytic trait structure does not become conserved among the CsAGOs proteins in *C*. *sinensis*.

**Table 2:** Comparison of the Argonaute proteins with missing catalytic residue(s) in PIWI domains between *C*. *sinensis* and *A*. *thaliana*.

Serial No.	*Citrus sinensis*		*Arabidopsis thaliana*^*b*^	
	Argonaute	Motifs^a^	Argonaute	Motifs^a^
1	CsAGO1	DDH/H	AtAGO1	DDH/H
2	CsAGO4	DDH/P	AtAGO4	DDH/S
3	CsAGO5a	DDY/H	AtAGO5	DDH/H
4	CsAGO5b	D_ _ /R	AtAGO6	DDH/P
5	CsAGO5c	DDY/P	AtAGO7	DDH/H
6	CsAGO6	DDH/S	AtAGO10	DDH/H
7	CsAGO7	DDH/H		
8	CsAGO10	DDH/H		

^a^Comparison of conserved motif corresponds to D760, D845, H986/H798 of Arabidopsis AGO1; D: aspartate, H: histidine, P: proline, R: arginine, S: serine, Y: tyrosine and “_” represents the missing catalytic residue(s).

^b^From Kapoor et al. (2008)

Surprisingly, the histidine residue at the 786^th^ position was replaced by proline (P) in CsAGO4 and CsAGO5c; by arginine (R) in CsAGO5b and in CsAGO6, H786 residue was replaced by serine (S) residue (Table 2). Due to the replacement of the conserved DDH/H motif residues in the reported CsAGO proteins, it can be assumed that the newly identified amino acid residues in the metal-chelating catalytic triad positions (DDH/H) may appear for genetic diversification or natural mutation. These changes indicate that the correspondent CsAGO proteins may fail to perform the endonuclease cleavage activities or the newly introduced residues may reflect new significant biological function in *C*. *sinensis* that can be explored through the expression analysis of the reported genes. Therefore, more expression analysis is required to investigate the functionality of the PIWI domain with the new catalytic residues in *C*. *sinensis*. Besides, two catalytic residues are missed in CsAGO5b protein but not in CsAGO5a although they are paralogous and the chromosomal location are in the same scaffold. The pI values of the CsAGOs indicated that the proteins have the basic properties as the pI values are greater than 7 and above 9.

The newly identified 4 CsRDR proteins that shared a common domain RdRP which consist of a sequence motif corresponds to the catalytic β' subunit of DNA-dependent RNA polymerases [86]. The *CsRDRs* have the genome length varying from 4373 bp to 11526 bp corresponding coding potentiality of 1157 and 1015 amino acids for CsRDR6 (orange1.1g041430m) and CSRDR3 (orange1.1g001771m) protein, respectively. In *C*. *sinensis*, no CsRDR4 and CsRDR5 candidates were identified in this analysis in comparison with the *A*. *thaliana*. The gene encoding ORF length was varied from 2715 to 3594 bp corresponding to *CsRDR1* and *CsRDR6*, respectively. The previous study reported that more RDR proteins (CsRDR1b/c; CsRDR6b) were found as subgroup members of CsRDR1(a/b/c) and the CsRDR6(a/b) while CsRDR4/5 types were not found [46]. The identified *CsRDRs* genes were close orthologues of the *AtRDRs* in structures, evaluation and characteristics found in *C*. *sinensis*.

### Phylogenetic analysis of DCL, AGO and RDR proteins in *C*. *sinensis* and *A*. *thaliana*

**Fig 3:**
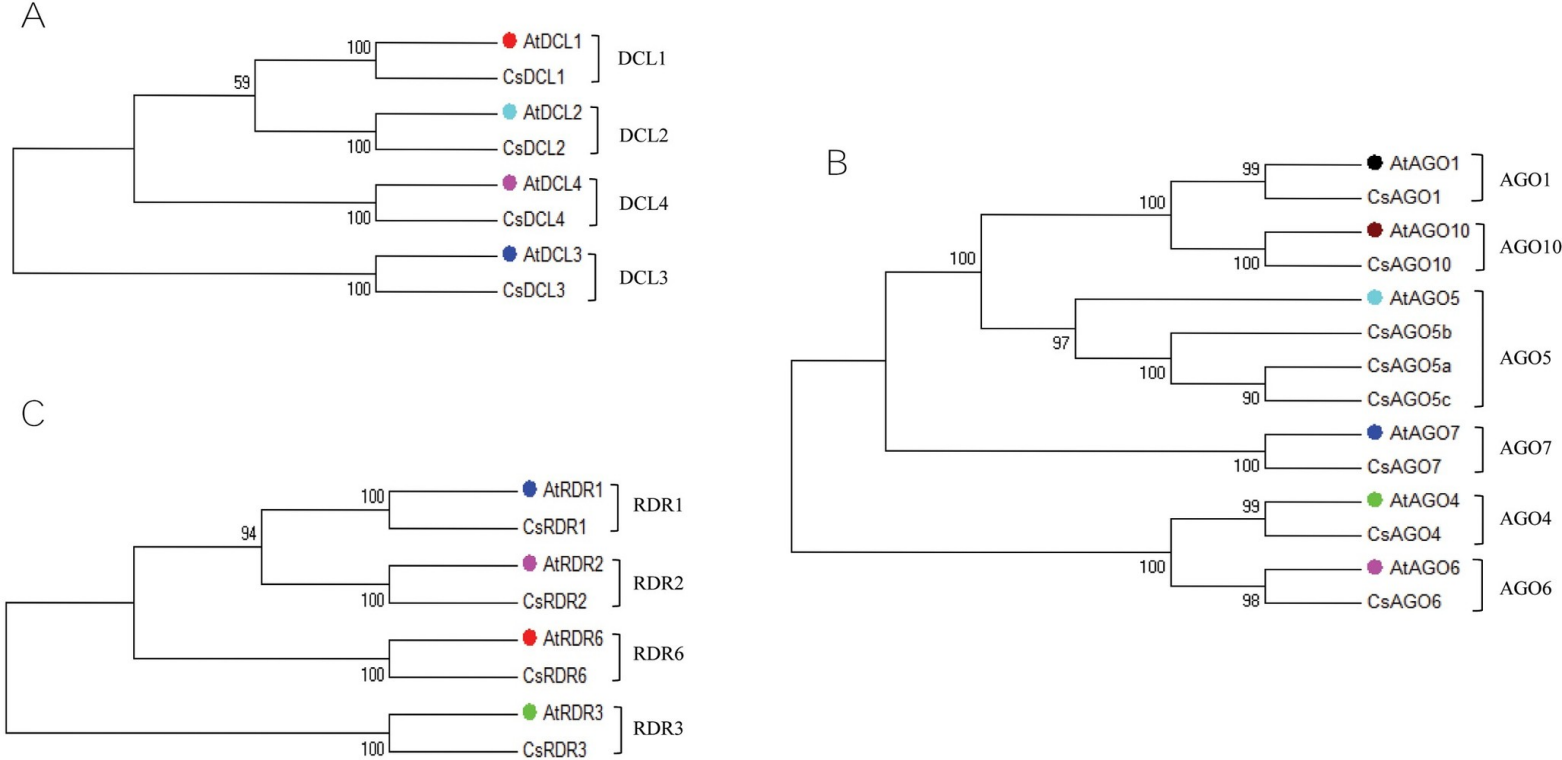
Phylogenetic tree for the (A) Dicer-like (DCL) proteins (B) Argonaute (AGO) proteins and (C) RDR proteins from *C*. *sinensis* and *A*. *thaliana*. All the phylogenetic trees were constructed by neighbour-joining method considering significant bootstrap values. The accession number and the abbreviations of proteins from *A*. *thaliana* are given below while others are tabulated in (Table 1): (A) Four DCL proteins, AtDCL1 (At1g01040), AtDCL2 (At3g03300), AtDCL3 (At3g43920) and AtDCL4 (At5g20320) were used in DCL analysis. (B) AtAGO1 (At1g48410), AtAGO4 (At2g27040), AtAGO5 (At2g27880), AtAGO6 (At2g32940), AtAGO7 (At1g69440) and AtAGO10 (At5g43810) were considered for AGO analysis. (C) Among six AtRDR proteins, the phylogenetic tree exhibited only four major classes with CsRDR proteins. The RDR proteins from *A*. *thaliana* were AtRDR1 (At1g14790), AtRDR2 (At4g11130), AtRDR3 (At2g19910) and AtRDR6 (At3g49500). The three different gene families from *A*. *thaliana* are indicated by different colours in the constructed tree adjacent to the designation.

To investigate the phylogenetic relationship of *C*. *sinensis* RNAi related genes, phylogenetic tree was constructed for CsDCL, CsAGO and CsRDR proteins along with the candidate proteins of *A*. *thaliana*. Phylogenetic tree was generated from the full-length aligned protein sequences (S1 Data) of the 4 CsDCLs and 4 AtDCLs from *C*. *sinensis* and *A*. *thaliana* (Fig 3A). The four CsDCL proteins (CsDCL1-4) were divided into four subgroups along with the corresponding DCLs in *A*. *thaliana* (AtDCL1-4) with well-supported bootstrap values. The CsDCL proteins showed high sequence conservation with their corresponding counterpart in *A*. *thaliana*. Every DCL subfamily comprised a single CsDCL protein.

To construct the phylogenetic tree for CsAGO proteins, the full-length protein sequence of the 8 CsAGOs and 6 AtAGOs were considered (S2 Data). The tree exhibited six subfamilies, AGO1, AGO4, AGO5, AGO6, AGO7 and AGO10 with the AtAGOs (Fig 3B). The AGO1 subfamily consists only a single *C*. *sinensis* protein named CsAGO1 with the *A*. *thaliana* AGO protein AtAGO1. Among the other AGO subfamilies, each showed a group containing a single *C*. *sinensis* AGO protein with a single *A*. *thaliana* AGO protein, except AGO5 cluster.

The AGO5 subfamily included three *C*. *sinensis* proteins with a single *A*. *thaliana* protein AtAGO5, which were named CsAGO5a, CsAGO5b and CsAGO5c on the basis of higher sequence similarity to AtAGO5. In CsAGO5 group, three paralogs were identified in *C*. *sinensis* when the CsAGO5a/b were located in similar scaffold location having unique genomic structures. The AGO1, AGO4, AGO6, AGO7 and AGO10 groups exhibited each separated cluster with a single AGO protein from *C*. *sinensis* and a single protein from *A*. *thaliana*.

Four main classes of *RDR* genes in *C*. *sinensis* were revealed by the phylogenetic analysis of the full-length protein sequences (S3 Data) of RDR proteins of *C*. *sinensis* and *A*. *thaliana* (Fig 3C). The CsRDR proteins were designated as CsRDR1, CsRDR2, CsRDR3 and CsRDR6 corresponding to the RDR proteins of Arabidopsis AtRDR1, AtRDR2, AtRDR3 and AtRDR6, respectively, for the increased sequence similarity. The predicted CsRDR proteins were clustered according to their high sequence conservation with their reflection part in *A*. *thaliana* RDR proteins.

The number of DCL, AGO and RDR proteins are conserved in different species that may or may not be similar. For instance, 4 DCLs for *Arabidopsis*, 8 for rice, millet, and *B*. *napus*, 5 for Barley, 4 for sugarcane and many more were reported for different monocots and dicot species [14, 40–42, 44, 45]. The number of identified AGO proteins exhibited a wide range of clades across the plant lineage. The maximum number of AGO proteins and their clades were observed in the flowering plants, for example, 22 AGO proteins in soybean (*Glycine max*, a paleopolyploid) [87], 21 in sugarcane [45], 19 in rice [14], and 17 in maize [40]. Although the genomic diversity exists among the AGO proteins in flowering plant, the three common clades can be observed through phylogenetic analysis: the AGO1/5/10, AGO2/3/7, and AGO4/6/8/9 clades [88]. The reported CsAGO proteins also contained at least one member for each of the significant clades. On the other hand, the number of *RDR* gene family members varied from minimum 5 [14, 40, 41, 89] to maximum 16 [38]. In our analysis, we have identified four *CsRDR* genes, while *CsRDR4/5* were found to be absent in *C*. *sinensis* genome indicating their evolutionary structure and functional effectiveness may be substituted or changed in *C*. *sinensis*. The *DCL4* and *AGO1* are two common and effective RNAi related genes found in all monocots and dicots [45] that have also been identified in our analysis.

### Conserved domain and motifs analysis of predicted proteins

The functional conserved domains of the predicted CsDCLs, CsAGOs and CsRDRs were retrieved by conserved domain searching databases Pfam, NCBI-CDD and Simple Modular Architecture Research Tool (SMART) analysis. The summary results are tabulated in Table 3.

**Table 3:** Domain analysis of the DCLs, AGOs and RDRs proteins of the predicted gene mapping on *C*. *sinensis* with Pfam, SMART and NCBI-CDD.

Serial Number	Gene Name	Accession Number	Domains		
			Pfam	SMART	NCBI-CDD
*CsDCLs*					
1	CsDCL1	orange1.1g000174m	DEAD, Helicase_C, Dicer_dimer, PAZ, Ribonuclease_3, Ribonuclease_3, dsrm, DND1_DSRM	DEXDc, Helicase_C, Dicer_dimer, PAZ, RIBOc, RIBOc, DSRM, DSRM	PAZ super family, helicase_C, Rnc, RIBOc, Dicer_dimer, DSRM
2	CsDCL2	orange1.1g000607m	DEAD, Helicase_C, Dicer_dimer, PAZ, Ribonuclease_3, Ribonuclease_3	DEXDc, Helicase_C, Dicer_dimer, PAZ, RIBOc, RIBOc	helicase_C, RIBOc, PAZ super family, Dicer_dimer, RIBOc
3	CsDCL3	orange1.1g000379m	ResIII, Helicase_C, Dicer_dimer, PAZ, Ribonuclease_3	DEXDc, Helicase_C, Dicer_dimer, Low complexity PAZ, RIBOc	helicase_C, PAZ super family, RIBOc, Dicer_dimer, Rnc super family
4	CsDCL4	orange1.1g000380m	DEAD, Helicase_C, Dicer_dimer, PAZ, Ribonuclease_3, Ribonuclease_3, DND1_DSRM	DEXDc, Helicase_C, Dicer_dimer, PAZ, RIBOc, RIBOc, DSRM, DSRM	helicase_C, RIBOc, Dicer_dimer, RIBOc, PAZ super family, DSRM
*CsAGOs*					
1	CsAGO1	orange1.1g001466m	Gly-rich_Ago1, ArgoN, ArgoL1, PAZ, ArgoL2, ArgoMid, Piwi	Gly-rich_Ago1, ArgoN, DUF1785, ArgoL1, PAZ, ArgoL2, ArgoMid, Piwi	Piwi-like super family, Gly-rich_Ago1
2	CsAGO4	orange1.1g002449m	ArgoN, ArgoL1, PAZ, ArgoL2, ArgoMid, Piwi	ArgoN, DUF1785, PAZ, ArgoL2, ArgoMid, Piwi	Piwi-like super family
3	CsAGO5a	orange1.1g002204m	ArgoN, ArgoL1, PAZ, ArgoL2, ArgoMid, Piwi	ArgoN, DUF1785, PAZ, ArgoL2, ArgoMid, Piwi	Piwi-like super family
4	CsAGO5b	orange1.1g037086m	PAZ, ArgoL2, ArgoMid, Piwi	PAZ, ArgoL2, ArgoMid, Piwi	Piwi-like super family, PAZ
5	CsAGO5c	orange1.1g003630m	ArgoN, ArgoL1, PAZ, ArgoL2, ArgoMid, Piwi	ArgoN, DUF1785, PAZ, ArgoMid, Piwi	Piwi-like super family
6	CsAGO6	orange1.1g002661m	ArgoN, ArgoL1, PAZ, ArgoL2, Piwi	ArgoN, DUF1785, PAZ, ArgoL2, Piwi	Piwi-like super family
7	CsAGO7	orange1.1g001684m	ArgoN, ArgoL1, PAZ, Piwi	ArgoN, DUF1785, PAZ, ArgoMid, Piwi	Piwi_ago-like, PAZ, ArgoL1, ArgoN
8	CsAGO10	orange1.1g001954m	ArgoN, ArgoL1, PAZ, ArgoL2, ArgoMid, Piwi	ArgoN, DUF1785, PAZ, ArgoL2, ArgoMid, Piwi	Piwi-like super family
*CsRDRs*					
1	CsRDR1	orange1.1g002586m	RdRP	RdRP	RdRP
2	CsRDR2	orange1.1g001183m	RdRP	RdRP	RdRP, RRM_SF super family
3	CsRDR3	orange1.1g001771m	RdRP	RdRP	RdRP
4	CsRDR6	orange1.1g041430m	RdRP	RdRP	RdRP

**Fig 4:**
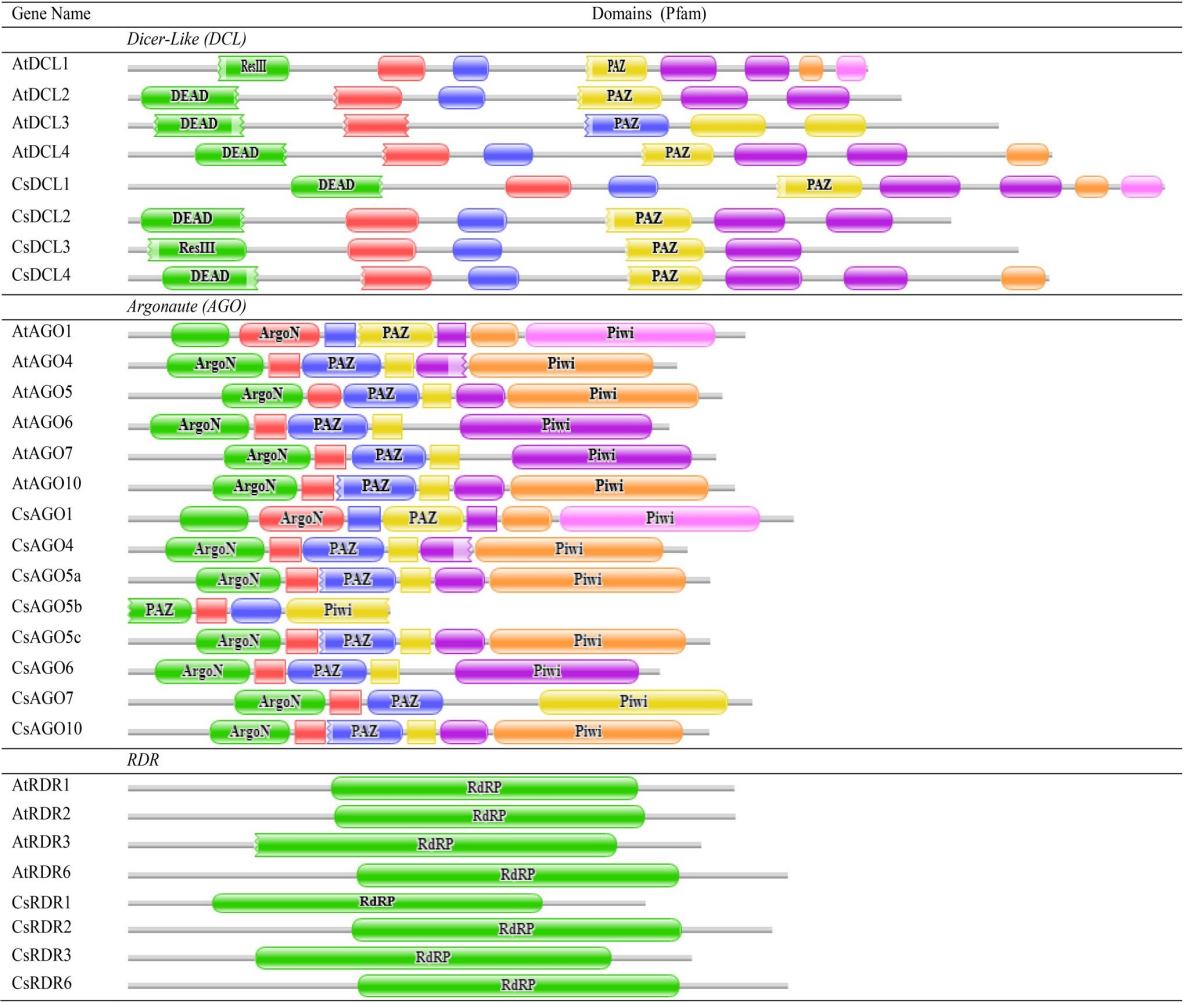
The conserved domains of the predicted proteins were drawn by using Pfam database.

The CsDCLs proteins showed all the conserved domains through the SMART analysis and also exhibited some unknown regions and low complexity regions besides the expected domains (Fig 4).

From the conserved domain search results from the Pfam database, NCBI database and the SMART analysis, all proteins reflected that half of the predicted DCL proteins (CsDCL1 and CsDCL4) were conserved with the DEAD/ResIII, Helicase_C, Dicer-dimer, PAZ, RNase III and dsRM domains, which were preserved by all the plant DCL proteins from the DCL genes family (class 3 RNase III family) [90, 91]. On the other hand, CsDCL2 and CsDCL3 have missed a single dsRM domain while others are contained with a second dsRM domain. The non-plant DCL proteins lacked the dsRM domain completely [23]. Compared to others DCL proteins, the CsDCL1 had the N-terminal DEAD domain which might consist of three adjacent segments of amino acid sequence within the full domain length (152 amino acids), resulted by the analysis of Pfam databases and SMART. The CsDCL3 also revealed the ResIII domain instead of the DEAD domain. Previous study revealed that the joint activities of the DCL2 and DCL3 are very important in disease response whereas DCL4 is also responsive in viral disease defense [92]. Therefore, the expression analysis of the reported *DCL* genes is necessary to explore their extensive biological role in *C*. *sinensis*.

The AGO proteins are the main elements for processing the double stand RNA in single stand and trigger the whole target RNA cleavage process. The findings showed that the PAZ and PIWI domain are the key functional domain for constructing RNA Induced Silencing Complex (RISC) in all species [14, 25, 26]. All the reported CsAGO proteins also preserved the other conserved domains like the *Arabidopsis* i.e. ArgoN, ArgoL1, DUF1785, ArgoL2. Moreover, the conserved domain ArgoMid was present in all the CsAGO proteins except the CsAGO6 (orange1.1g002661m) which does not contain any MID domain. The six of the 8 CsAGO proteins started with the ArgoN domain while only the CsAGO1 (orange1.1g001466m) started with the Gly-rich domain and the CsAGO5b (orange1.1g037086m) started with the PAZ domain. Although the CsAGO5b posed the PAZ, ArgoMid and the PIWI domain, it did not contain the common DUF1785 domain. This seems that the CsAGO5b is found as a novel member of the RNAi related gene family in *C*. *sinensis*. The identified CsDCL and CsAGO proteins also contain the opulent number of functional domains including the main functional domain as in *A*. *thaliana* and some additional regions (Table 3). The RNA dependent RNA polymerase (RdRp) is one of the most versatile enzymes of RNA viruses that is indispensable for replicating the genome as well as for carrying out transcription. All the reported CsRDR consistently posed the RdRp domain while the CsRDR2 showed the RRM_SF super family region, found from NCBI-CDD analysis. The putatively functional *RDR1* and *RDR2* genes have a significant impact on siRNA biogenesis and accelerate the RNAi process [93, 94].

By MEME-suite analysis, the DCLs proteins had 19 (in CsDCL2 and CsDCL3) and 20 (in CsDCL1 and CsDCL4) motifs among the 20 motifs as mentioned before. The predicted motifs were well distributed among the DCL domains for all CsDCLs proteins. The MEME analysis of CsAGOs proteins identified 16 common conserved motifs among all the AGO proteins from *C*. *sinensis* and *A*. *thaliana*, except the CsAGO5b having 9 conserved motifs. The predicted conserved motifs were distributed among the AGO domains in *C*. *sinensis* AGO proteins.

**Fig 5:**
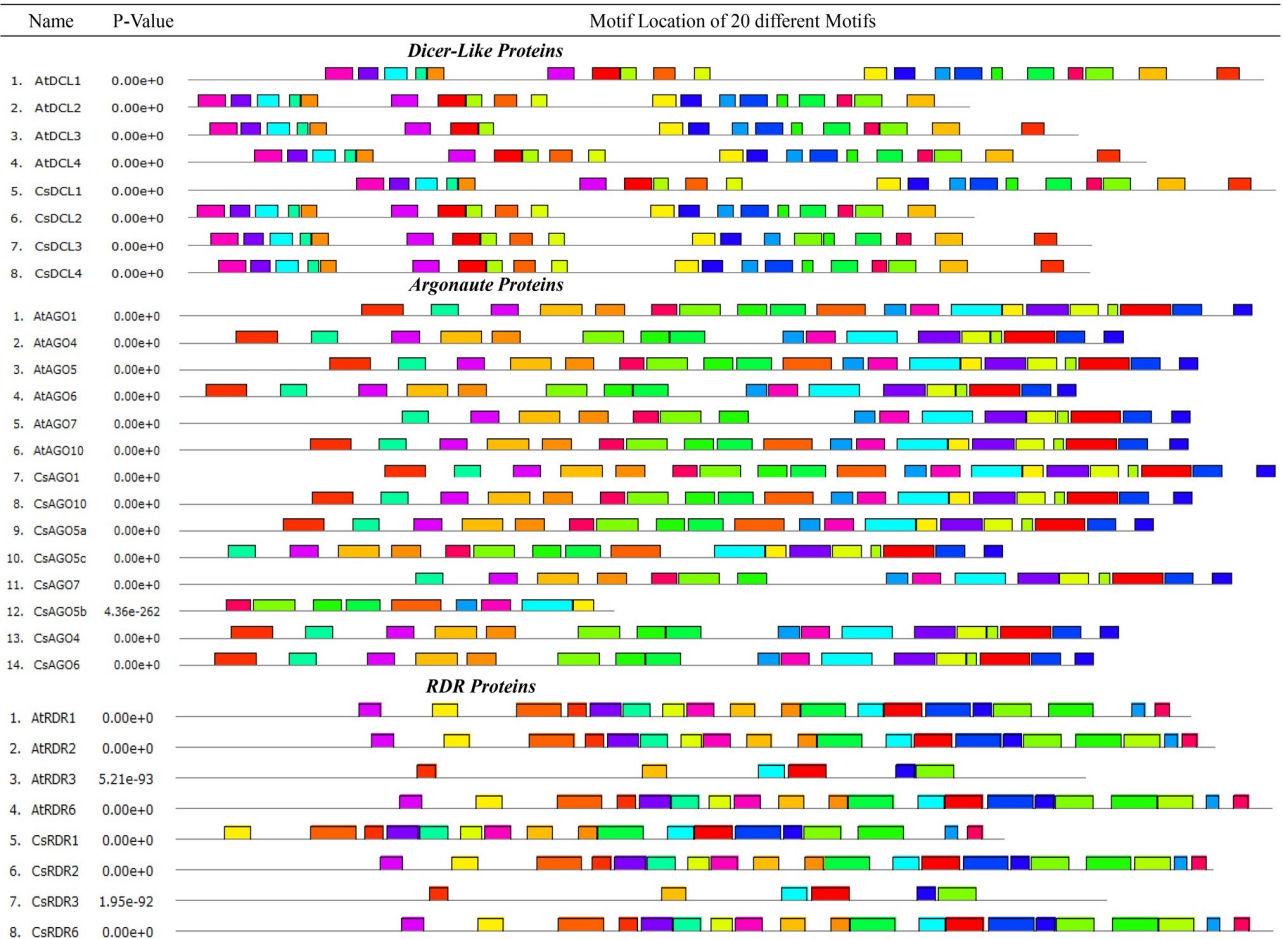
Conserved motifs of the proteins of different gene families are drawn by MEME-suite (maximum 20 motifs are displayed). Different colour represented various motifs distributed in the domains of the proteins.

Among the CsAGO proteins one had 16 different motifs (CsAGO7), three proteins (CsAGO4, CsAGO5c and CsAGO6) reflected 17 motifs and others three proteins (CsAGO1, CsAGO5a and CsAGO10) contained 20 conserved motifs (Fig 5). Although from the analysis it is observed that the conservation within the AGO proteins of *C*. *sinensis* and *A*. *thaliana* is balanced there still has some variability of motif distribution between the different subfamilies of AGO proteins in *C*. *sinensis*. Moreover, this analysis suggested that the conserved predicted motifs might play important roles in these AGO proteins.

In RDR protein family, the MEME analysis exhibited that the least 6 conserved motifs in CsRDR3 coincided with the AtRDR3. Among other CsRDRs proteins, the CsRDR2 and CsRDR6 presented 20 out of 20 conserved motifs which are well distributed on the RdRP domain and the CsRDR1 had 18 out of 20 conserved motifs (Fig 5). Although the predicted motifs were well conserved in the major part of the RDR domain, the motif schemes of different RDR subfamilies did not follow the same distributional pattern. The RDR proteins also reflected some additional motifs besides the RdRP domain having unknown functional role. However, the MEME-suite analysis reflected that the CsDCLs, CsAGOs and CsRDRs proteins were enriched with balanced conservation and distribution of the motifs throughout the subfamilies. This analysis suggested that the multiple functional domains and predicted motifs might play vital roles in the functional importance of these genes in *C*. *sinensis* in different developmental stages which can be investigated through expression analysis.

### Gene structure and genomic location of *CsDCLs*, *CsAGOs* and *CsRDRs*

**Fig 6:**
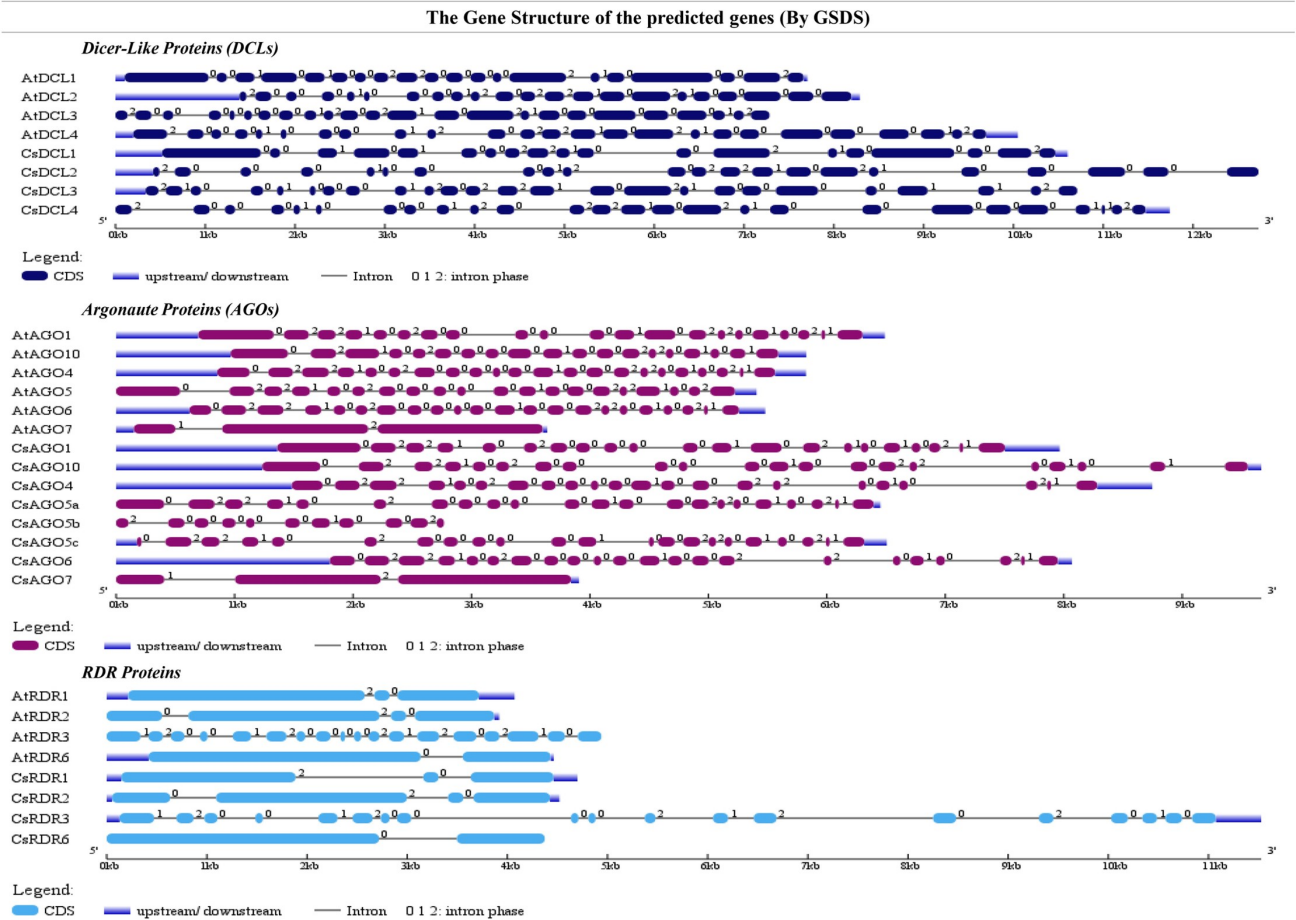
Gene structure of the predicted *CsDCLs*, *CsAGOs* and *CsRDRs* genes in *C*. *sinensis* with *A*. *thaliana* using Gene Structure Display Server (GSDS 2.0, http://gsds.cbi.pku.edu.cn/index.php) [77].

To observe the gene structure of the predicted *CsDCLs*, *CsAGOs* and *CsRDRs* gene, their exon-intron configuration was explored by using GSDS with the respective genes family of *A*. *thaliana*. The exon-intron configuration of the predicted genes represented higher conservation as expected for that of *DCLs*, *AGOs* and *RDRs* genes in the model plant *A*. *thaliana* (Fig 6). The gene structure of *CsDCLs* exhibited having the number of intron 18–25 (Table 1), bearing higher similarity with *AtDCLs*.

On the other hand, out of eight *CsAGOs*, six genes displayed 20 or 21 introns in the gene structure except the *CsAGO5b* and *CsAGO7* having the number of introns 10 and 2, respectively, (Fig 6). This structure indicated that *CsAGOs* genes are highly similar to the *AtAGOs*. The *CsRDRs* showed up with the equal number of introns with their orthologs from *A*. *thaliana*, except *CsRDR3*, which, having 18 introns, is just one short of that in *AtRDR3*.

**Fig 7:**
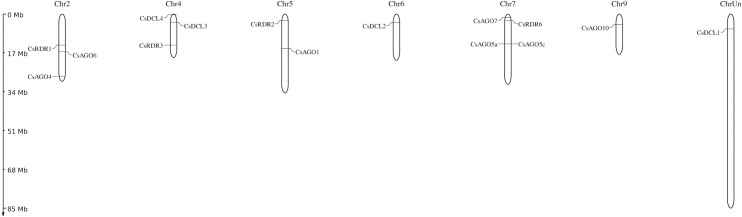
The genomic location of the reported *CsDCL*, *CsAGO* and *CsRDR* genes. The chromosomal length indicating scale is provided on the left. The ChrUn means the unknown chromosome.

The genomic location of the predicted RNAi pathway related genes in *C*. *sinensis* was conducted by observing the position of the genes in different scaffold location. The predicted *CsDCLs*, *CsAGOs* and *CsRDRs* genes were distributed among the 15 different scaffolds through the entire genome (S1 Table). All genes had a unique scaffold position while the two *CsAGO* genes (*CsAGO5a* and *CsAGO5b*) were placed in the scafold000595 in different location. Furthermore, the chromosomal location was constructed and analyzed to check the genomic distribution of the reported genes (Fig 7). The identified RNAi related genes were scattered among the chromosomes of the *C*. *sinensis* when none of them were located in the chromosome 1, 3 and 8. The *CsDCL3* and *CsDCL4* were distributed on the chromosome 4 when the chromosome 6 and the unknown chromosome contained the *CsDCL2* and *CsDCL1* gene only. Among the *CsAGO* genes, 5 genes were found in the chromosome 2 (*CsAGO4/5)* and chromosome 7 (*CsAGO5a/5c/7)*.

The *CsAGO1* and *CsAGO10* appeared in the chromosome 5 and 10 separately (Fig 7). In the chromosome 7, two paralogous of the *CSAGO5* (*CsAGO5a/c*) were neighboring in very close genomic location indicating a higher possibility of tandem duplication. Also, the *CsAGO7* and *CsRDR6* were located closely in chromosome 7. Therefore, it can be pre-assumed that these genes will perform a diverse expression pattern due to their appearance that can be studied further under various condition and stresses. The four *CsRDR* genes are scattered in chromosome 2, 4, 5, and 7 (Fig 7).

### Gene ontology enrichment analyses

**Fig 8:**
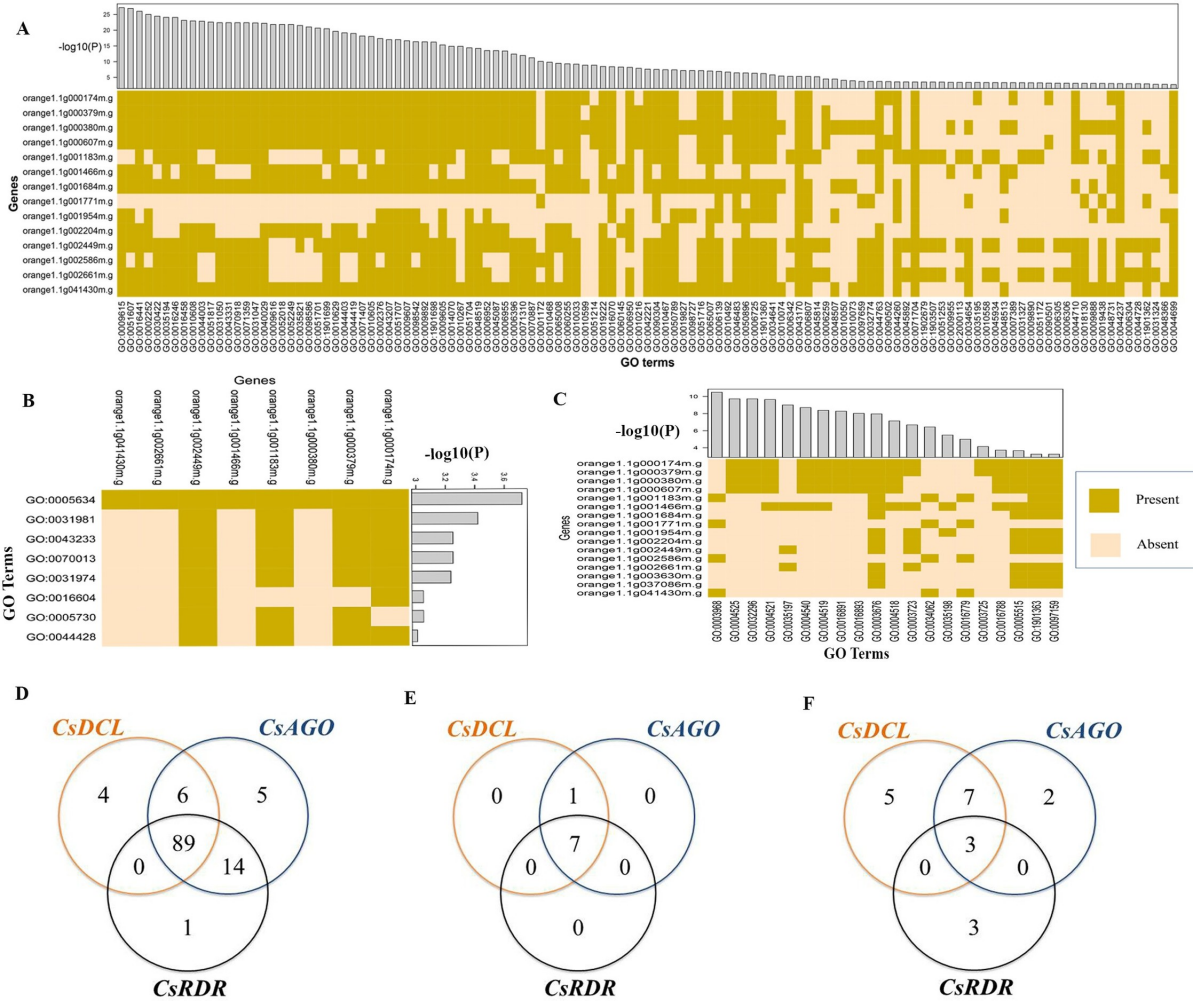
The heatmap for the predicted GO terms corresponding to the reported RNAi genes are presented for (A) biological process (B) cellular components (C) molecular function whether the genes are related (Present) or not (Absent). The p-value corresponding to the GO terms are showed in histogram adjacent to the heatmap, using -log10 (*p*-value). The Ven diagrams are drawn to observe the shared GO terms by three gene families considering the (D) biological process (E) cellular components (F) molecular functions.

The Gene Ontology (GO) analysis predicts the location or functional similarity of genes within the cells that are over- or under-expressed where the information are gathered from literature, database and computational evidences [95, 96]. The different GO terms describes the engagement with the various functional pathways linked to the reported genes. In order to better understand the biological roles of the predicted RNAi pathway related genes and characterize them, GO enrichment analysis was performed (Fig 8 and S4 Data) from the PlantTFDB. From the analysis result it was observed that of the reported genes 12 were involved in post-transcriptional gene silencing (PTGS) pathway (GO: 0016441; *p*-value: 3.60e-27), 10 were related to RNA interference (GO: 0016246; *p*-value: 8.50e-25) and 12 genes were associated with gene silencing (GO: 0016458; *p*-value: 7.40e-24). The RNAi is closely related to the phenomenon named post-transcriptional gene silencing (PTGS) in plants [97]. As most of the predicted genes significantly showed the GO terms those are associated with the RNAi, it clearly indicates that these genes have a great involvement with mRNA degradation process in the *C*. *sinensis*.

The GO enrichment analysis showed that five predicted RNAi pathway genes (among 16) were related to the endonuclease activity (GO: 0004519; *p*-value = 4.20e-09) which (S4 Data) indicates a positive linkage with the RNA-induced silencing complex (RISC) mediated cleavage activities into the cell. This multimeric protein complex (i.e. RISC) guides protein degradation. Among the RNAi proteins, Argonautes work for the cleavage called endonucleolytic activities, which result in the final PTGS for specific mRNA substrate [98]. The *CsAGOs* may also have the various biological activities that can be revealed by their expression analysis against any biological question. There were 13 genes related to nucleic acid binding (GO: 0003676; *p*-value = 1.10e-08), 7 genes to RNA binding (GO: 0003723; *p*-value = 2.10e-07) and 12 genes related to protein binding (GO: 0005515; *p*-value = 0.00022) activities (S4 Data) which indicate the RNAi protein’s participation to the RISC as well as interference processes conducted. The predicted CsAGO proteins contained the special domains called PAZ and PIWI domain that play the key role in making a complex with RNA or DNA. The PAZ domain has a nucleic acid-binding fold that promotes the domain to bind to the specific position of the nucleic acids [99, 100] by binding with the target mRNA for degradation. The GO enrichment analysis also showed the attachment of the predicted genes to the numerous biological processes. Significantly, most of the reported genes were engaged with the regulation of biological process (GO: 0050789; *p*-value = 3.90e-08), negative regulation of gene expression (GO: 0010629; *p*-value: 2.30e-20) and dsRNA fragmentation (GO: 0031050; *p*-value: 4.10e-23) (S4 Data) which are also parts of the greater RNAi process.

The *C*. *sinensis* RNAi candidate genes were also involved in virus response (GO: 0009615; *p*-value: 6.70e-28), immune response (GO: 0006955; *p*-value: 4.10e-14) as these were reported for *AtDCL* and *AtRDR* [11, 23, 35, 49, 55–57]. These GO enrichment analysis for biological processes (S1A Fig), molecular functions (S1B Fig) and cellular component (S1C Fig) undoubtedly indicated that the predicted genes are deeply interrelated with the RNAi pathway in *C*. *sinensis*. In addition, the predicted genes act with the hydrolase activity, acting on ester bond that was predicted from the GO analysis (S4 Data).

The Ven Diagram of the GO terms for three clusters of the RNAi associated genes was drawn (Fig 8). It was observed that the *CsDCL*, *CsAGO* and *CsRDR* genes had significant number of GO pathways in common. In biological process, there were 89 GO enrichment pathways (Fig 8) were shared by the reported proteins, which indicate the involvement of the RNAi gene members in numerous biological processes together in *C*. *sinensis*. Also, in molecular function and cellular component, the predicted genes exhibited a group of mutual GO pathways. So, this GO analysis provides a significant indication of the predicted RNAi member genes in this study.

### Sub-cellular localization of the reported genes and proteins

**Fig 9:**
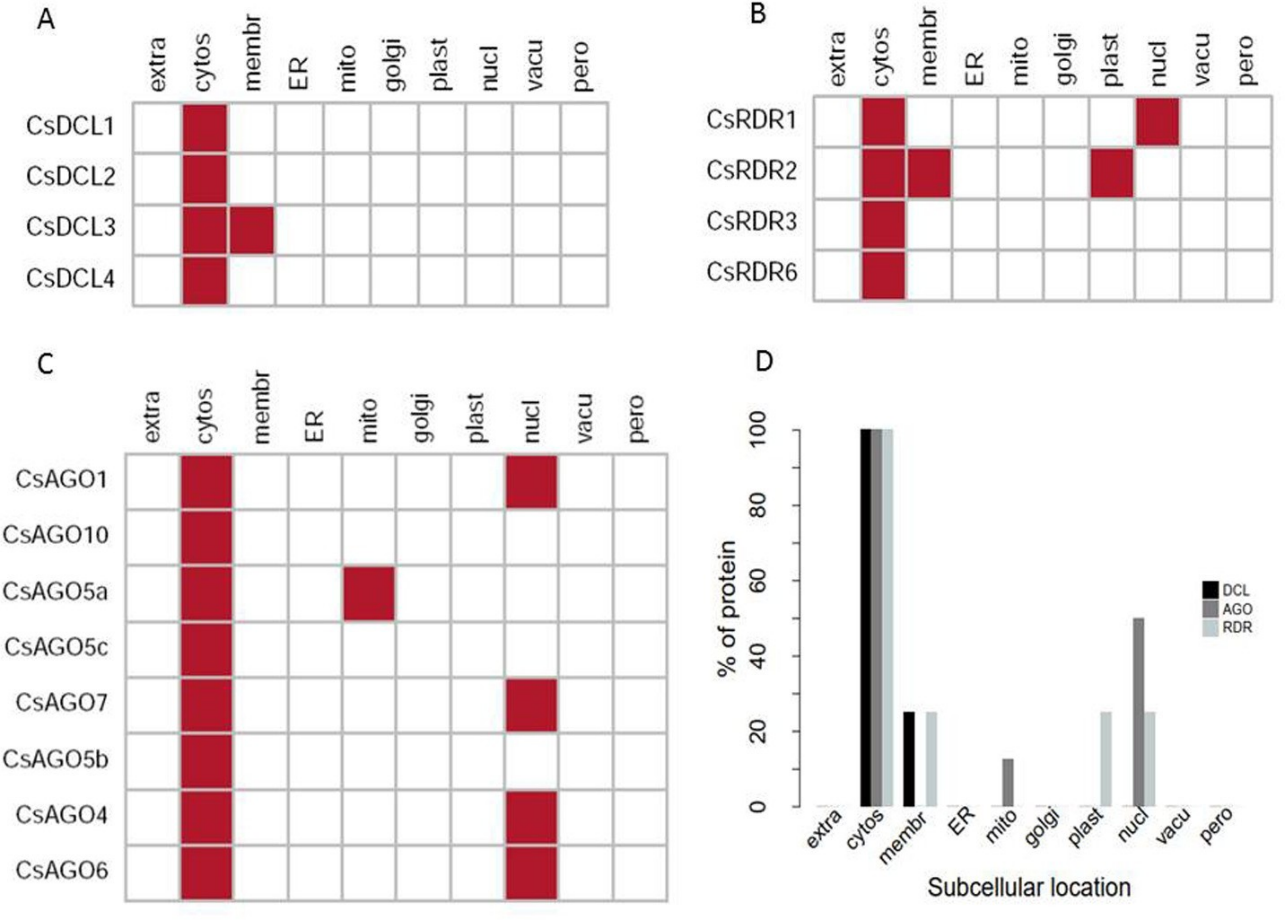
Sub-cellular localization analysis for (A) CsDCL (B) CsRDR and (C) CsAGO proteins. (D) The percentage of protein appears in different cellular components. Here cytosol (cytos), endoplasmic reticulum (ER), extracellular (extra), golgi apparatus (golgi), membrane (membr), mitochondria (mito), nuclear (nucl), peroxisome (pero), plastid (plast) and vacuole (vacu). Overall report is tabulated in (S2 Table).

The subcellular localization studies of the predicted proteins were observed to uncover their cellular appearance. By the sub-cellular localization annotation, it has been shown that all the predicted proteins reported in this study appear into the cytosol (Fig 9). As PTGS occurs into the cytoplasmic region [101], this result implies that the reported RNAi proteins may directly involve to the PTGS process. On the other hand, four CsAGO and one CsRDR proteins exhibited their appearance into the nucleus whereas no CsDCLs were located there. These bring a significant importance whether the CsDCLs are not found in nucleus. Further expression pattern analysis will provide deeper insight about the *CsDCLs*. Some of the identified RNAi proteins were also distributed into the cell membrane, plastid and mitochondria (Fig 9).

Previous studies reported that the RNAi genes are not only highly related with PTGS but also with transcriptional gene silencing (TGS) [101]. In protein transcriptional process, RNA polymerase type II complexes are directly involved [102]. For PTGS, the RNAi proteins have greater participation in RNA-induced silencing complex (RISC) mediated cleavage activities by the help of DCL, AGO and RDR proteins with other molecules [98]. The PTGS happens into the cytoplasmic region for targeted mRNA protein degradation [102].

### Regulatory relationship between TFs and RNAi genes

Transcription factors (TFs) play the central roles as drivers of gene expression in all living organisms since they control the rate of genetic transcription and coordinate the action of any genetic network [103, 104]. The studies evidence that the various family of TFs are associated with the growth and development of the aboveground and underground parts of plants, abiotic stress responses, response to pathogens and many more [103, 105–109]. Thus, identification of the regulatory TFs of the reported RNAi genes can help to improve our understanding of gene silencing process in *C*. *sinensis*. In this analysis a total of 235 TFs those regulate the predicted RNAi genes (S5 Data) were identified. The identified TFs were distributed into 27 groups based on the TF families. The TFs MYB, Dof, ERF, NAC, MIKC_MADS, WRKY and bZIP families may play significant role in regulating RNAi genes. Particularly those of ERF, NAC, WRKY and bZIP families, which are the top four families, contained 29, 20, 20 and 10 TFs respectively, and accounted 57.66% of the total identified TFs (S3 Table). This finding indicates that those TFs can be important in regulating RNAi genes.

**Fig 10:**
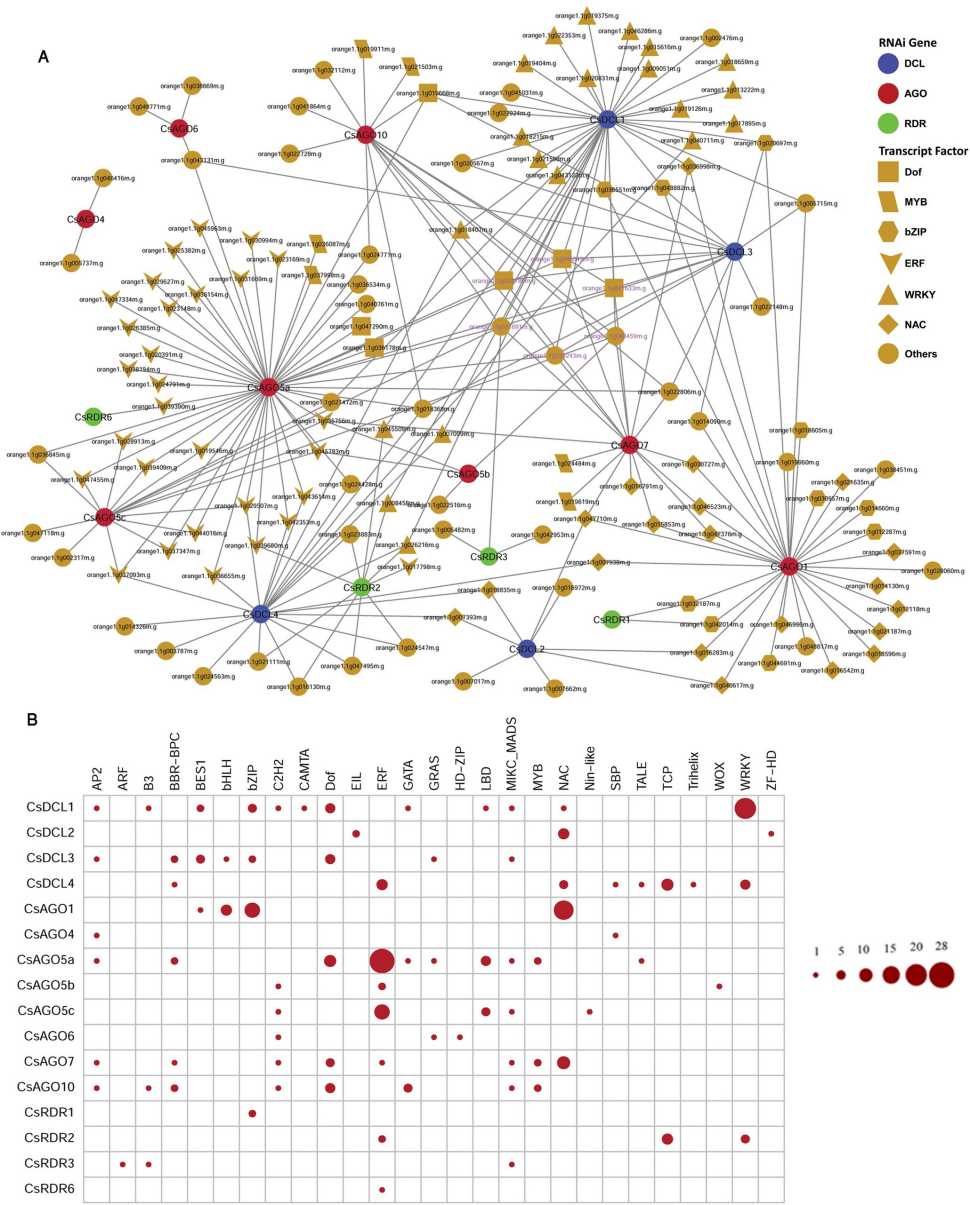
(A) The regulatory network among the TFs and the predicted RNAi genes. The nodes of the network were coloured based on RNAi genes and TFs. *DCL*, *AGO* and *RDR* genes were represented by blue, red and green node colour, respectively, and the TFs were represented by yellow node colour. Different node symbols were used for different families of TFs. Magenta node level was used for the hub TFs. (B) The map representing the associated number of TFs with the *CsRNAi* genes.

**Fig 11:**
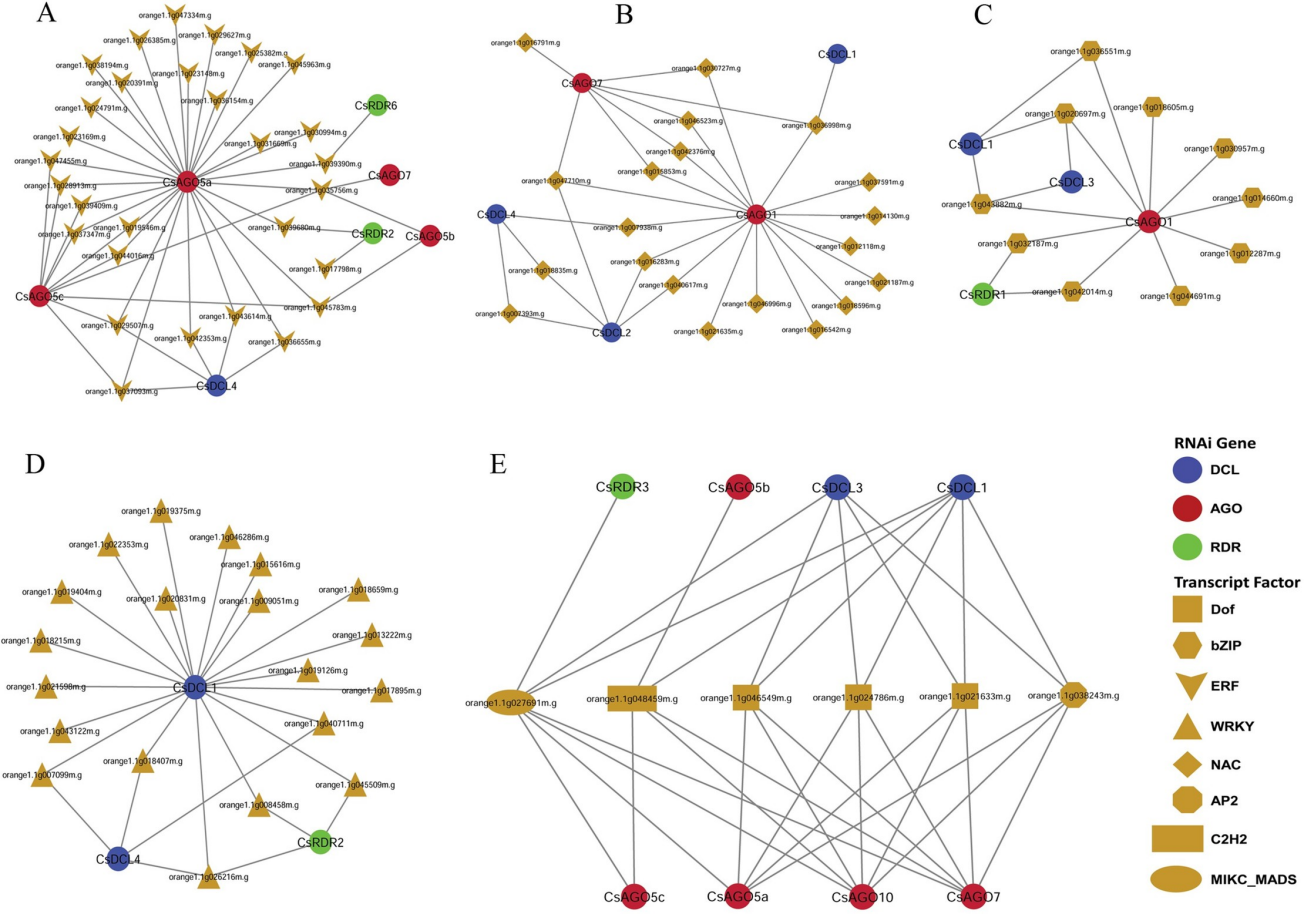
RNAi gene mediated sub-network for (A) ERF, (B) NAC, (C) bZIP, and (D) WRKY TF family. (E) Sub-network among the hub TFs those regulate more than five RNAi genes.

From the resultant network it was observed that different groups of TFs exhibited distinct structure. For example, TFs belonging to ERF family mainly linked to the gene *CsAGO5a* (Fig 10B and S2 Fig). However, some RNAi genes such as *CsAGO5c*, *CsDCL4* and others were also regulated by ERF family; all of them were also linked to *CsAGO5a* (Fig 11A). Very similar results were also observed for the hub TFs NAC, WRKY and bZIP (Fig 11B–11D).

Moreover, six hub TFs were identified on the basis of node degree which had more than five interacting partners with the RNAi related genes (Fig 11E). All of the hubs TFs were connected to eight RNAi genes. Out of eight RNAi genes corresponding to hub TFs, five are *AGO*, two are *DCL* and only one is *RDR*. Three RNAi genes (2 *AGOs*: *CsAGO10*, *CsAGO7*; 1 DCL: *CsDCL1*) were predicted to be regulated by the entire six hub TFs. Among them 3 belonged to Dof TFs family and other three were in MIKC_MSDS, C2H2 and bZIP (Fig 11E). The Dof TFs family is directly involved with the DNA binding activities by the N- and C-terminal region and causes the regulation of gene activation or repression of the target genes which is the main theme of RNAi. The Dof TFs family also works for the biosynthesis of flavonoids and glucosinolates, stress tolerance, seed germination and controlling the photoperiodic flowering [110–114]. The MYB TFs are available in both plants and animals which contain MYB domain (a 52 amino acid motif) [115]. The MYB TF plays a significant role in biotic and abiotic response in Arabidopsis [116, 117]. The expression patterns of the WRKY family of TFs are associated with the defence response against biotrophic pathogens, necrotrophic pathogens and also works as anti-microbial defence [118–120]. Moreover, the expression of the RDR gene families may be influenced by the MYB, NAC and the WRKY TFs during various stress conditions and they have a direct or indirect involvement in plant development and stress response in plant [115, 120–123]. The expression of the defensive genes is regulated through the interaction activities of Calmodulin (CaM) with the specific TFs MYB, NAC and the WRKY [124, 125]. Our study indicates that the further gene expression study needs to clarify whether the calcium/CaM related pathways are playing a vital role in RNAi-related pathways in *C*. *sinensis* or not. From the network analysis it is observed that the MIKC_MADS (orange1.1g027691m) TF regulates maximum seven RNAi related genes and the rest of the TFs regulate five RNAi genes (Fig 11E). This MIKC_MADS TF family also involves with the transcription of *OsRDR1* genes to augment the tolerance power against the *Rice stripe virus (RSV)* in rice (*Oryza sativa*) [126]. The regulatory network clearly exposes that these predicted genes and the associated TFs of RNAi process in *C*. *sinensis* will exhibit a wide ranges expression pattern that can be retrieved by deeper investigation of these genes in future.

### *Cis*-acting regulatory element analysis

**Fig 12:**
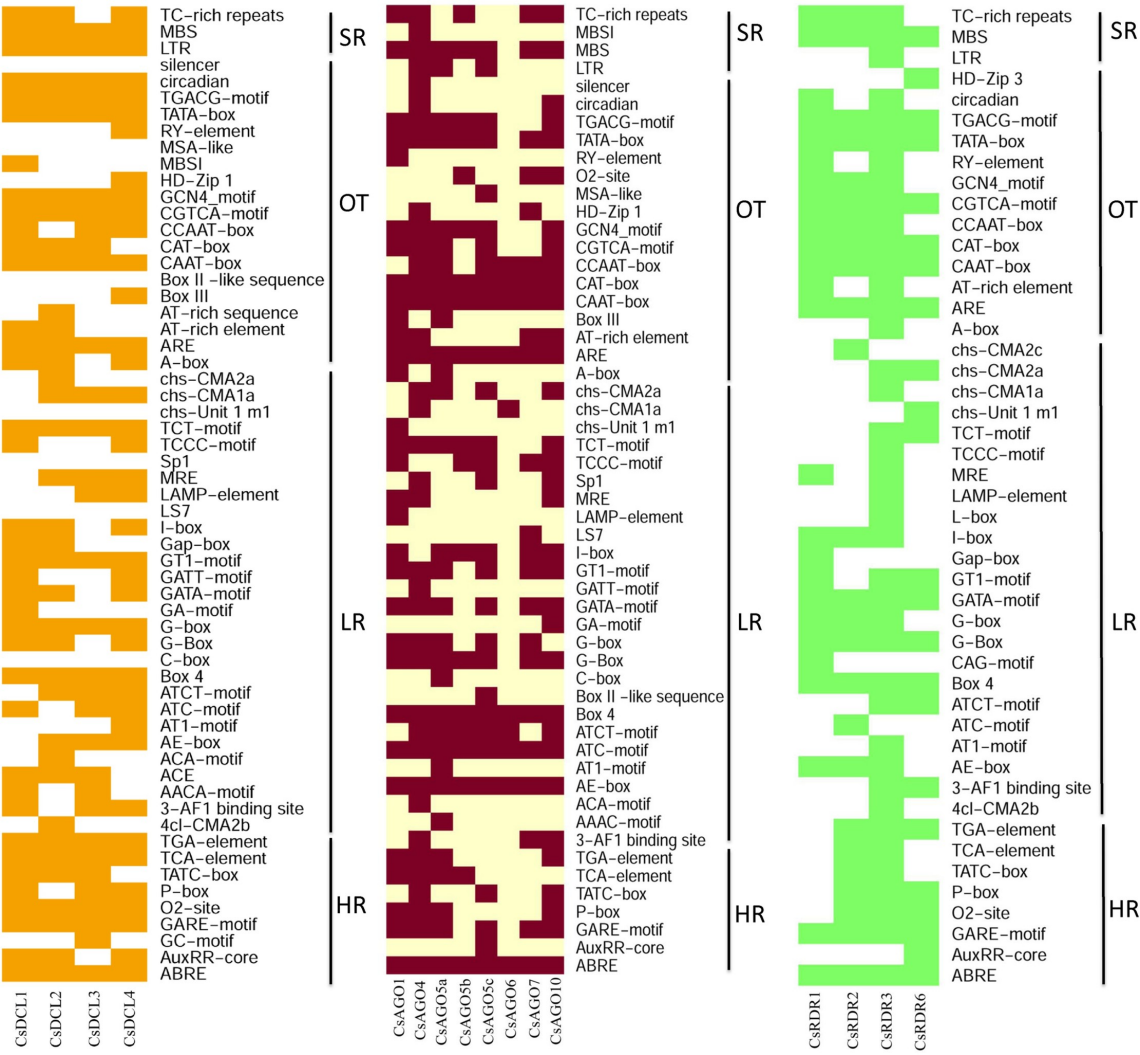
The *cis*-regulatory element in the promoter region of the identified *C*. *sinenis DCLs*, *AGOs* and *RDRs* genes, respectively. The deep color represents the presence of that element with the corresponding genes.

The *cis*-acting regulatory elements (CAREs) are short motifs (5±20 bp) where the TFs can bind to the specific targeted genes to initiate the transcription and control gene regulation process [127]. The *cis*-elements are also involved in plant defence response, stress responsiveness [127, 128]. The wet lab experimental exploration of these inevitable regulatory component is technically challenging and expensive whether their computational identification is being used through various enriched databases [127]. The *cis*-acting regulatory element analyses were conducted to find out the functional diversity of the motifs related to the promoter region of the proposed RNAi genes into *C*. *sinenesis*. The PlantCARE database provided the information about the motifs and their functionality with the genes. The analysis revealed that most of the motifs were light responsive (LR) (Fig 12), widely present in the entire RNAi gene’s promoter. Supporting the EST analysis later, the light responsiveness is associated with the photosynthesis which occurs in leaves. Among the light responsive motifs, the ATCT-motif, ATC-motif, Box-4, AE-box, G-box, I-box, GAT-motif, GT1-motif were shared by the most of the RNAi related genes in *C*. *sinensis* (Fig 12) [127, 129–132]. The TC-rich repeats (*cis*-acting element involved in defense and stress responsiveness) [133], MBS (MYB binding site involved in drought-inducibility) [134] and LTR elements (*cis*-acting element involved in low-temperature responsiveness) were commonly found as stress responsive motif among the *CsDCL*/*AOG*/*RDR* genes in *C*. *sinensis*.

It is known that the plant hormones are essential for plant growth and development. The significant plant hormone responsive (HR) *cis*-acting elements were identified in this analysis. The ABRE (*cis*-acting element involved in the abscisic acid responsiveness) [135, 136], AuxRR-core (*cis*-acting regulatory element involved in auxin responsiveness) [137, 138], GC-motif (enhancer-like element involved in anoxic specific inducibility) [139–141], GARE-motif (gibberellin-responsive element), O2-site (*cis*-acting regulatory element involved in zein metabolism regulation) [137, 138], P-box (gibberellin-responsive element), TATC-box (*cis*-acting element involved in gibberellin-responsiveness) [137, 142], TCA-element (*cis*-acting element involved in salicylic acid responsiveness) and TGA-element (auxin-responsive element) [138, 142, 143] were the hormone responsive *cis*-elements shared by the *CsDCLs*, *CsAGOs* and *CsRDRs* as phytohormones responsive element (Fig 12). There were some others significant elements identified and represented as others activities. The AT-rich element (binding site of AT-rich DNA binding protein (ATBP-1)), AT-rich sequence (element for maximal elicitor-mediated activation), CAAT-box (common *cis*-acting element in promoter and enhancer regions), CAT-box (*cis*-acting regulatory element related to meristem expression), CCAAT-box (MYBHv1 binding site), GCN4_motif (*cis*-regulatory element involved in endosperm expression), TATA-box (core promoter element around -30 of transcription start), circadian (*cis*-acting regulatory element involved in circadian control), silencer (GT-1 factor binding site) and TGACG-motif (*cis*-acting regulatory element involved in the MeJA-responsiveness) [129, 138, 142–144] were recognized as other *cis*-acting regulatory elements shared by RNAi related genes in *C*. *sinensis* (Fig 12). Some unknown *cis*-regulatory elements were also detected along with the reported *cis*-elements (S6 Data). In general, the *cis*-regulatory elements carried out significant evidences about the proposed RNAi genes that will be helpful for further investigation about their role in plant disease response, growth and development.

### *In silico* Expressed Sequence Tag (EST) analysis

**Fig 13:**
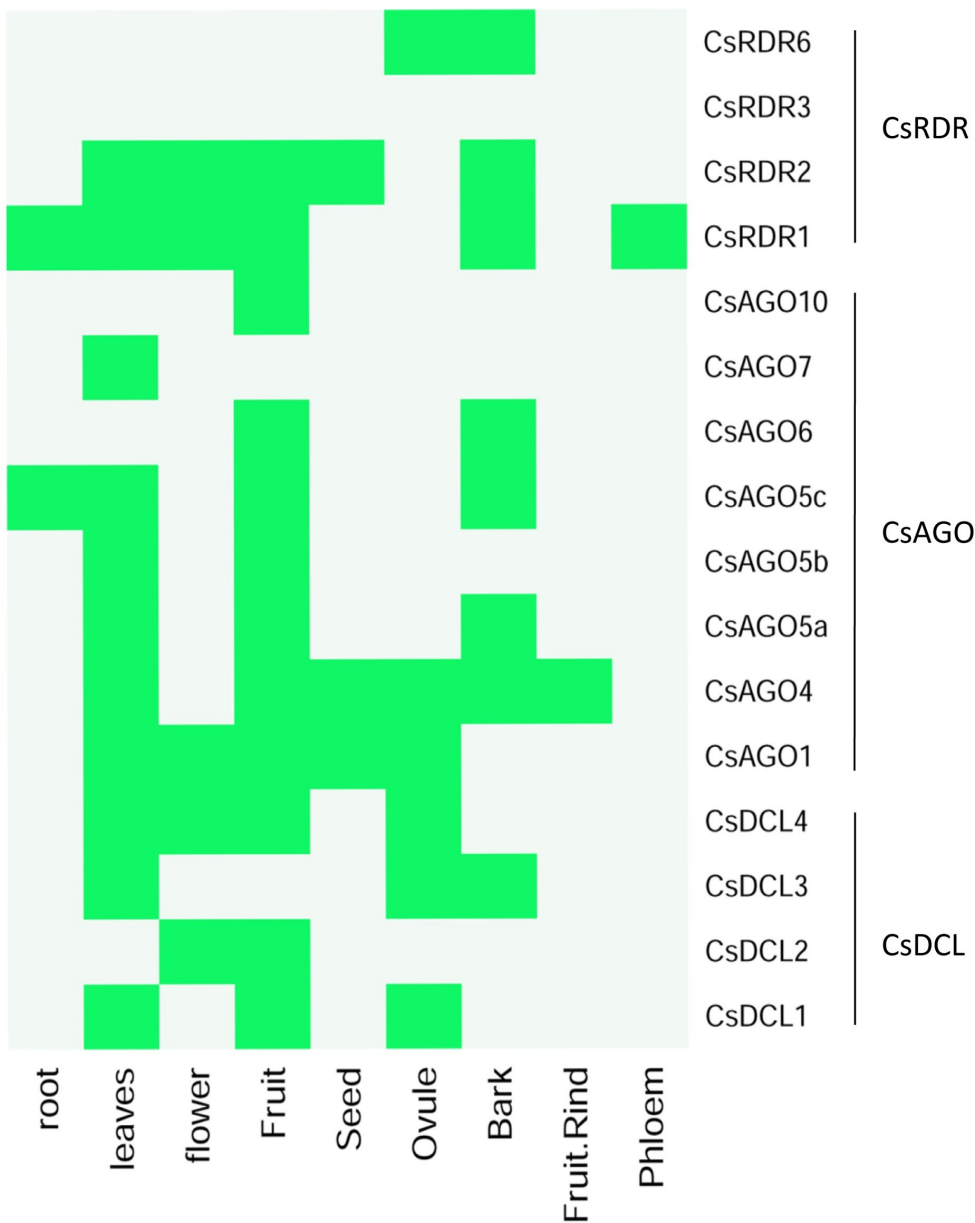
The *in silico* Expressed Sequence Tag (EST) analysis of the identified RNAi genes in *C*. *sinensis* plant. The green color represents the existence of expression and off color stands for absent of expression.

The EST mining results obtained from the PlantGDB database indicated that the sweet orange RNAi associated genes are expressed in multiple important tissues and organs. The search results provided 154 unique EST contigs records against the reported RNAi related genes of sweet orange. The GeneBank accession ID of the obtained EST contigs has been supplied in supplementary data file (S7 Data). However, evidence of expression of RNAi pathway related genes in several plant species showed their expression in leaf, root, flower, seeds and other organs [14, 33, 39–43, 82, 145, 146]. A recent study identified and characterized the expression of sweet orange RNAi related genes in several tissues and organs using RNA-seq data [46]. In general, most of the genes were expressed in leaf and fruit indicating their direct involvement in the photosynthesis and fruit developmental stages in *C*. *sinensis* (Fig 13).

Among the identified EST contigs, the expression of *CsDCLs* were detected in leaves (*CsDCL1*/*3*/*4*), flowers (*CsDCL2*/*4*), fruit (*CsDCL1*/*2*/*4*), ovule (*CsDCL1*/*3*/*4*) and bark (*CsDCL3*), and no expression were found in root. The entire *CsAGOs* exhibited diverse expression pattern in all the organs (roots, leaves, flowers, ovule, fruit, fruit rind and seeds) of sweet orange (Fig 13). Among the *CsAGO* genes, EST contigs of *CsAGO1* and *CsAGO4* were detected in most of the organs in *C*. *sinensis* while only the *CsAGO1*/*4* provided the confirmation of expression in seeds. Similarly, almost all the *CsRDRs* expressed in leaf, flower and fruit when the *CsRDR6* showed expression in ovule and bark. Although for the proposed *C*. *sinensis* RNAi related genes showed that all the genes have their expression at least in one organ or tissue, no evidence of expression were found for the *CsRDR3* in this *in silico* EST analysis (Fig 13). Collectively, the EST analyses indicated that the proposed RNAi related genes have vast contribution in ovule fertilization, fruit development process, plant photosynthesis which can be validated by tissue specific expression and functional study.

## Conclusion

The sweet orange is considered as the second highest produced fruits all over the world. The *C*. s*inensis* plant is the major source of sweet orange which is one of the most favourite and nutritious fruits. *In silico* characterization, diversity analyses and regulatory process of the RNAi-related gene families were essential, since these families play a vital role for silencing of other targeted genes in plant. Our study attempted to identify the RNAi pathway genes, keeping their key transcriptional factors and regulatory elements in focus, in *C*. *sinensis* along with the genomic and physicochemical information of the predicted genes and their corresponding proteins. With the phylogenetic analysis, the subgroups of the three gene families were exhibited, the domains and motifs configuration and the gene structures revealed the maximum homogeneity with the respective gene family of *A*. *thaliana*. Moreover, the GO enrichment and subcellular localization analysis provided the final confirmation about the reported genes and protein which are the key factor of RNAi process in *C*. *sinensis*. In this analysis, we explored regulatory relationship network between TFs and proposed RNAi genes. Potential TFs and *cis*-acting regulatory elements involved in plant growth and development as well as controlling the gene expression or suppression related to RNAi process were identified. The expressed sequence tag (EST) analysis indicates that the reported RNAi-related genes have diverse involvement into the orange plant growth, development and flowering processes. Thus, the reported genes in this study may exhibit significant expression pattern under different stress conditions in various developmental stages of sweet orange. Therefore, our findings may provide a basis for further functional analysis of RNAi pathway genes in *C*. *sinensis* to clarify their roles in growth, development, disease resistance and improve production and quality of sweet orange.

## Supporting information

S1 Table Gene location in different scaffold of the reported genes. (PDF)

S2 Table Sub-cellular localization of the predicted proteins. (PDF)

S3 Table Distribution of TF families those regulating RNAi genes. (PDF)

S1 Data Protein sequences of the reported DCL genes of *Citrus sinensis*. (TXT)

S2 Data Protein sequences of the reported AGO genes of *Citrus sinensis*. (TXT)

S3 Data Protein sequences of the reported RDR genes of *Citrus sinensis*. (TXT)

S4 Data GO enrichment analysis result for predicted RNAi genes. (XLSX)

S5 Data List of transcript factors and their families regulating predicted RNAi genes. (XLSX)

S6 Data List of *cis*-regulatory element associated with the reported RNAi genes. (XLSX)

S7 Data (XLSX)

S1 Fig GO enrichment analysis of the predicted RNAi genes **(A)** biological process, **(B)** molecular function and **(C)** cellular process. In the directed acyclic graph (DAG) the downstream term corresponds to a subset of the upstream term. The significant (*p-*value < 0.05, FDR < 0.05) GO terms are in colored boxes (the degree of color saturation is positively correlated to the enrichment level of the GO term), and non-significant terms are in white boxes.
(PDF)

S2 Fig Distribution of TF families corresponding to genes. Rows of the figure represent the predicted RNAi genes and the columns represent the families of the TFs. The number indicates the TF families regulate the RNAi genes.
(PDF)

## Abbreviations

AGO: Argonaute
BLAST: Basic Local Alignment Search Tool
bp: base pair
DCL: Dicer-Like
GO: Gene Ontology
GSDS: Gene Structure Display Server
HMM: Hidden Markov Model
MEME: Multiple Em for Motif Elicitation
miRNA: microRNA
PTGS: post-transcriptional gene silencing
RDR: RNA, dependent RNA polymerase
RNAi: RNA interference
RISC: RNA-induced silencing complex
sRNA: small RNA
siRNA: short interfering RNA
TF: transcription factor
TGS: transcriptional gene silencing
