# Supplementary material for: In silico identification and characterization of AGO, DCL and RDR gene families and their associated regulatory elements in sweet orange (Citrus sinensis L.)
Source: PLoS One. 2020 Dec 21;15(12):e0228233. doi: 10.1371/journal.pone.0228233 (PMC7751981; doi:10.1371/journal.pone.0228233)
Supplement: S1 Table — (PDF) [file pone.0228233.s001.pdf]

S1 Table: Gene location in different scaffold of the reported genes.

Gene Name	Accession Number	location	Location in scaffolds (Phytozome)
CsDCLs			
CsDCL1	orange1.1g000174m	scaffold00001:3480331..3490933	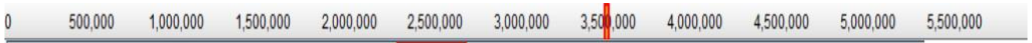
CsDCL2	orange1.1g000607m	scaffold00367:74912..87639	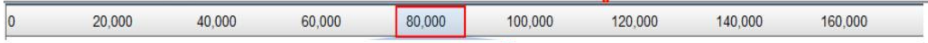
CsDCL3	orange1.1g000379m	scaffold00013:998934..1009643	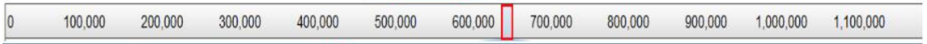
CsDCL4	orange1.1g000380m	scaffold00068:219006..230745	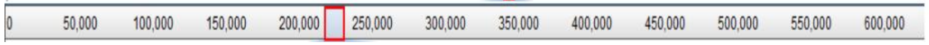
CsAGOs			
CcAGO1	orange1.1g001466m	scaffold00674:51962..59926	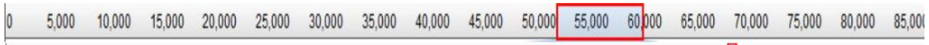
CcAGO4	orange1.1g002449m	scaffold00028:907375..916121	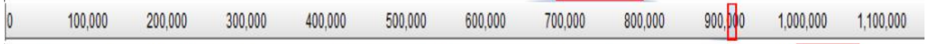
CcAGO5a	orange1.1g002204m	scaffold00595:85931..92382	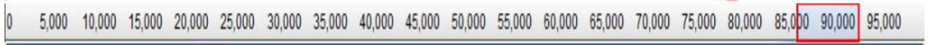
CcAGO5b	orange1.1g037086m	scaffold00595:96826..99593	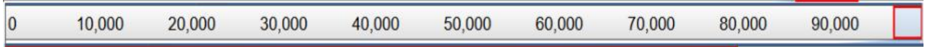
CcAGO5c	orange1.1g003630m	scaffold03700:12..6517	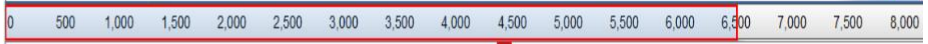
CcAGO6	orange1.1g002661m	scaffold00067:338566..346634	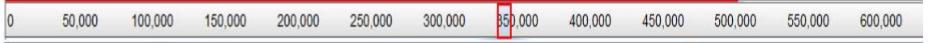
CcAGO7	orange1.1g001684m	scaffold00003:3309646..3313553	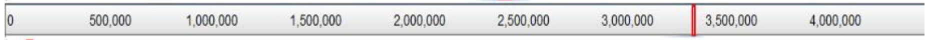
CcAGO10	orange1.1g001954m	scaffold00011:54251..63917	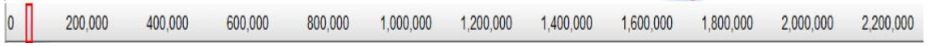
CsRDRs			
CsRDR1	orange1.1g002586m	scaffold00058:231382..236081	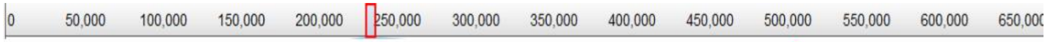
CsRDR2	orange1.1g001183m	scaffold00020:1255446..1250927	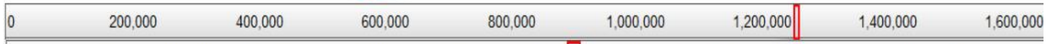
CsRDR3	orange1.1g001771m	scaffold00027:638984..650509	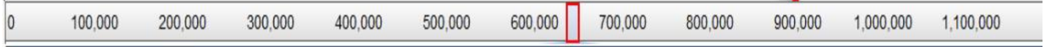
CsRDR6	orange1.1g041430m	Scaffold d00051:398116..402488	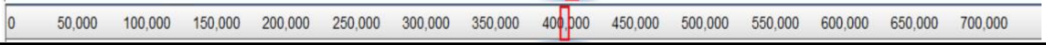
