# Supplementary material for: In silico identification and characterization of AGO, DCL and RDR gene families and their associated regulatory elements in sweet orange (Citrus sinensis L.)
Source: PLoS One. 2020 Dec 21;15(12):e0228233. doi: 10.1371/journal.pone.0228233 (PMC7751981; doi:10.1371/journal.pone.0228233)
Supplement: S2 Table — (PDF) [file pone.0228233.s002.pdf]

S2 Table: Sub-cellular Localization of the predicted proteins

	extra	cytos	membr	ER	mito	golgi	plast	nucl	vacu	pero
**CsDCL**	0	100	25	0	0	0	0	0	0	0
**CsAGO**	0	100	0	0	12.5	0	0	50	0	0
**CsRDR**	0	100	25	0	0	0	25	25	0	0

cytosol (cytos), endoplasmic reticulum (ER), extracellular (extra), golgi apparatus (golgi), membrane (membr), mitochondria (mito), nuclear (nucl), peroxisome (pero), plastid (plast) and vacuole (vacu).
