# Supplementary material for: In silico identification and characterization of AGO, DCL and RDR gene families and their associated regulatory elements in sweet orange (Citrus sinensis L.)
Source: PLoS One. 2020 Dec 21;15(12):e0228233. doi: 10.1371/journal.pone.0228233 (PMC7751981; doi:10.1371/journal.pone.0228233)
Supplement: S3 Table — (PDF) [file pone.0228233.s003.pdf]

S3 Table: Distribution of TF families those regulating RNAi genes.

TF Family	Count	Percent (%)
ERF	29	21.16788
NAC	20	14.59854
WRKY	20	14.59854
bZIP	10	7.29927
Dof	6	4.379562
MYB	6	4.379562
TCP	6	4.379562
bHLH	5	3.649635
LBD	5	3.649635
GATA	4	2.919708
B3	3	2.189781
BES1	3	2.189781
AP2	2	1.459854
BBR-BPC	2	1.459854
C2H2	2	1.459854
EIL	2	1.459854
SBP	2	1.459854
ARF	1	0.729927
CAMTA	1	0.729927
GRAS	1	0.729927
HD-ZIP	1	0.729927
MIKC_MADS	1	0.729927
Nin-like	1	0.729927
TALE	1	0.729927
Trihelix	1	0.729927
WOX	1	0.729927
ZF-HD	1	0.729927
