# Supplementary material for: In silico identification and characterization of AGO, DCL and RDR gene families and their associated regulatory elements in sweet orange (Citrus sinensis L.)
Source: PLoS One. 2020 Dec 21;15(12):e0228233. doi: 10.1371/journal.pone.0228233 (PMC7751981; doi:10.1371/journal.pone.0228233)
Supplement: S1 Fig — GO enrichment analysis of the predicted RNAi genes (A) biological process, (B) molecular function and (C) cellular process. In the directed acyclic graph (DAG) the downstream term corresponds to a subset of the upstream term. The significant (p-value < 0.05, FDR < 0.05) GO terms are in colored boxes (the degree of color saturation is positively correlated to the enrichment level of the GO term), and non-significant terms are in white boxes. (PDF) [file pone.0228233.s011.pdf]

(A)

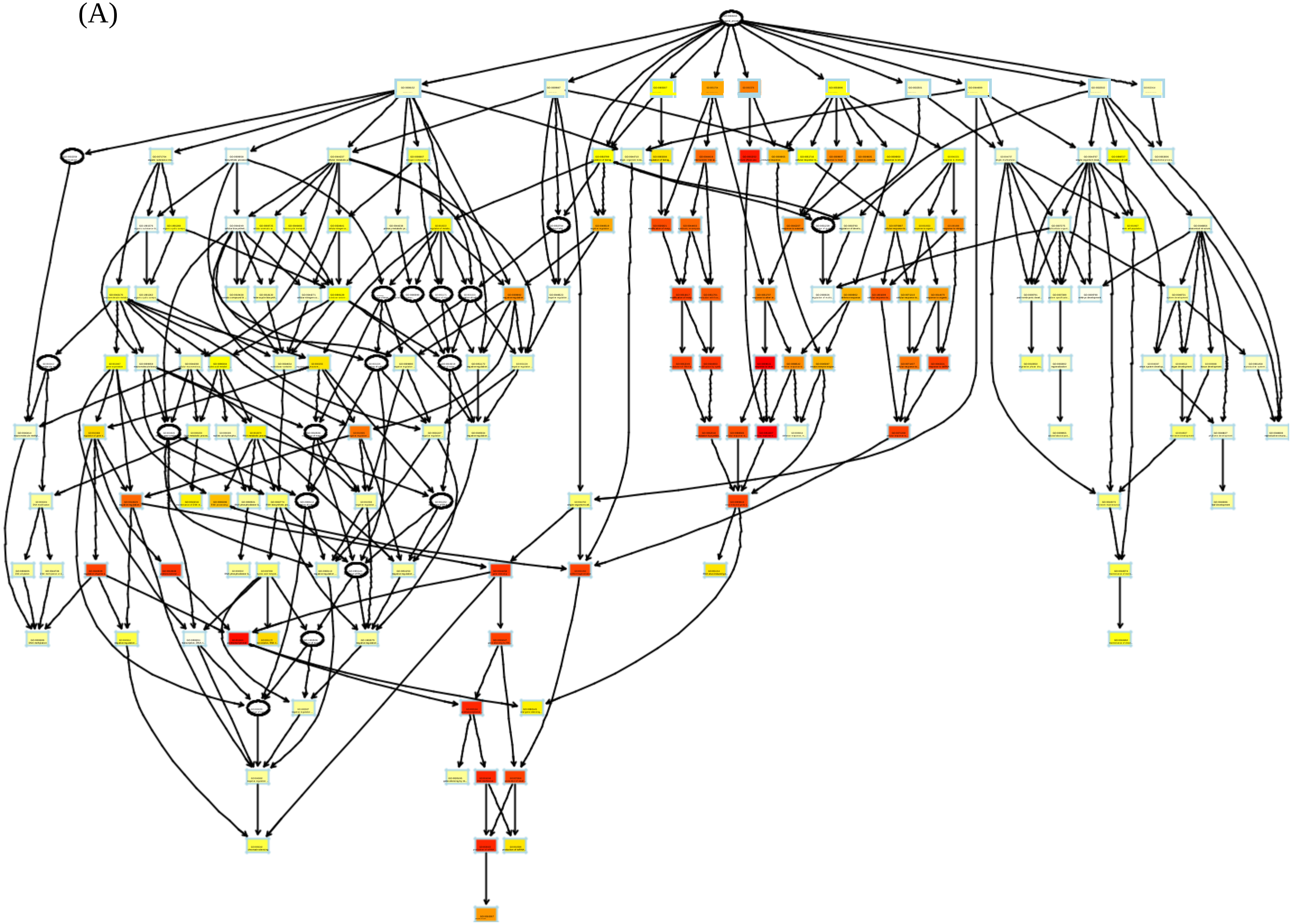

(B)

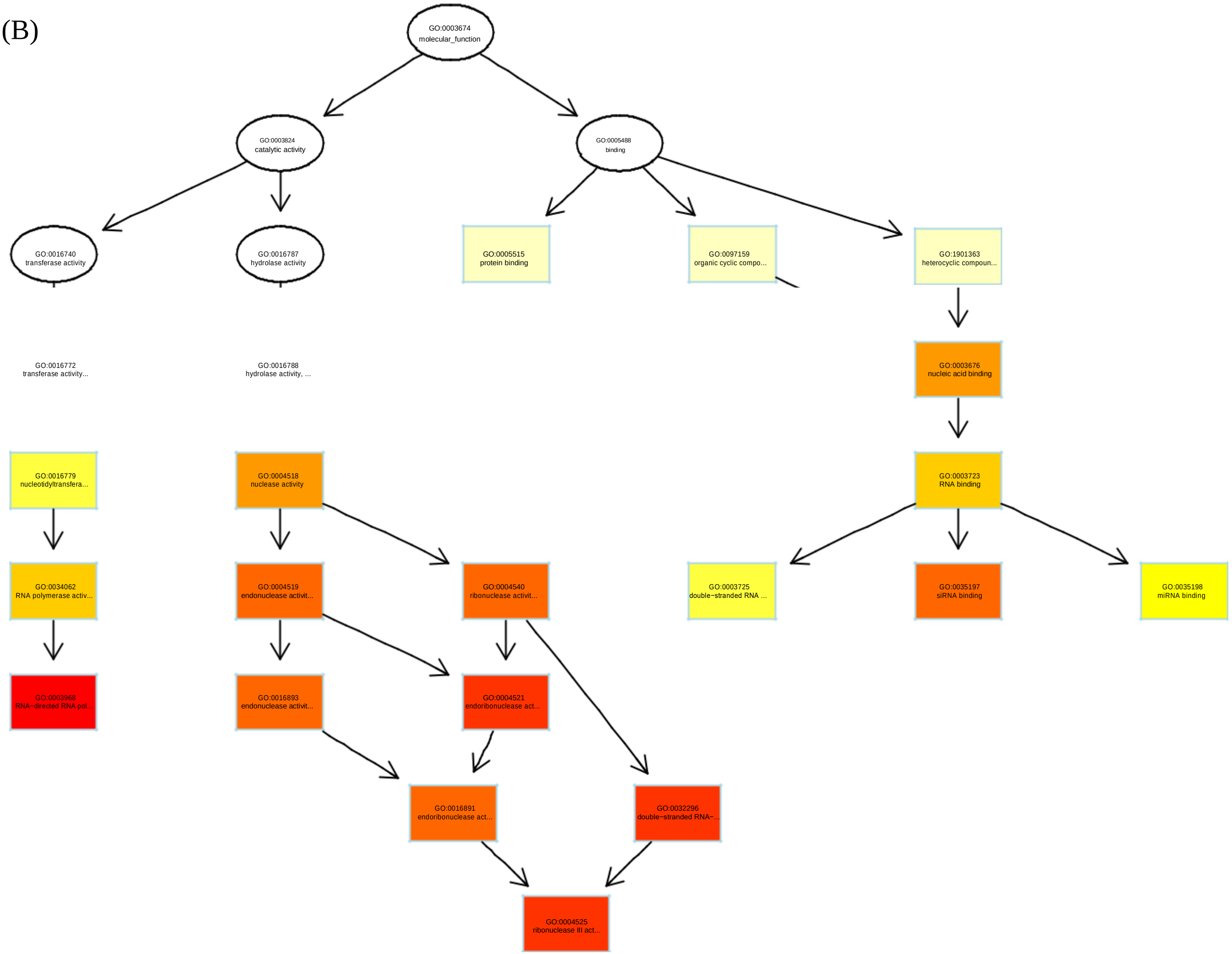
GO:0003674
molecular_function
GO:0003824
catalytic activity
GO:0005488
binding
GO:0016740
transferase activity
GO:0016787
hydrolase activity
GO:0005515
protein binding
GO:0097159
organic cyclic compo...
GO:1901363
heterocyclic compoun...
GO:0016772
transferase activity...
GO:0016788
hydrolase activity, ...
GO:0003676
nucleic acid binding
GO:0016779
nucleotidyltransfera...
GO:0004518
nuclease activity
GO:0003723
RNA binding
GO:0034062
RNA polymerase activ...
GO:0004519
endonuclease activit...
GO:0004540
ribonuclease activit...
GO:0003725
double−stranded RNA ...
GO:0035197
siRNA binding
GO:0035198
miRNA binding
GO:0003968
RNA−directed RNA pol...
GO:0016893
endonuclease activit...
GO:0004521
endoribonuclease act...
GO:0016891
endoribonuclease act...
GO:0032296
double−stranded RNA−...
GO:0004525
ribonuclease III act...

(C)

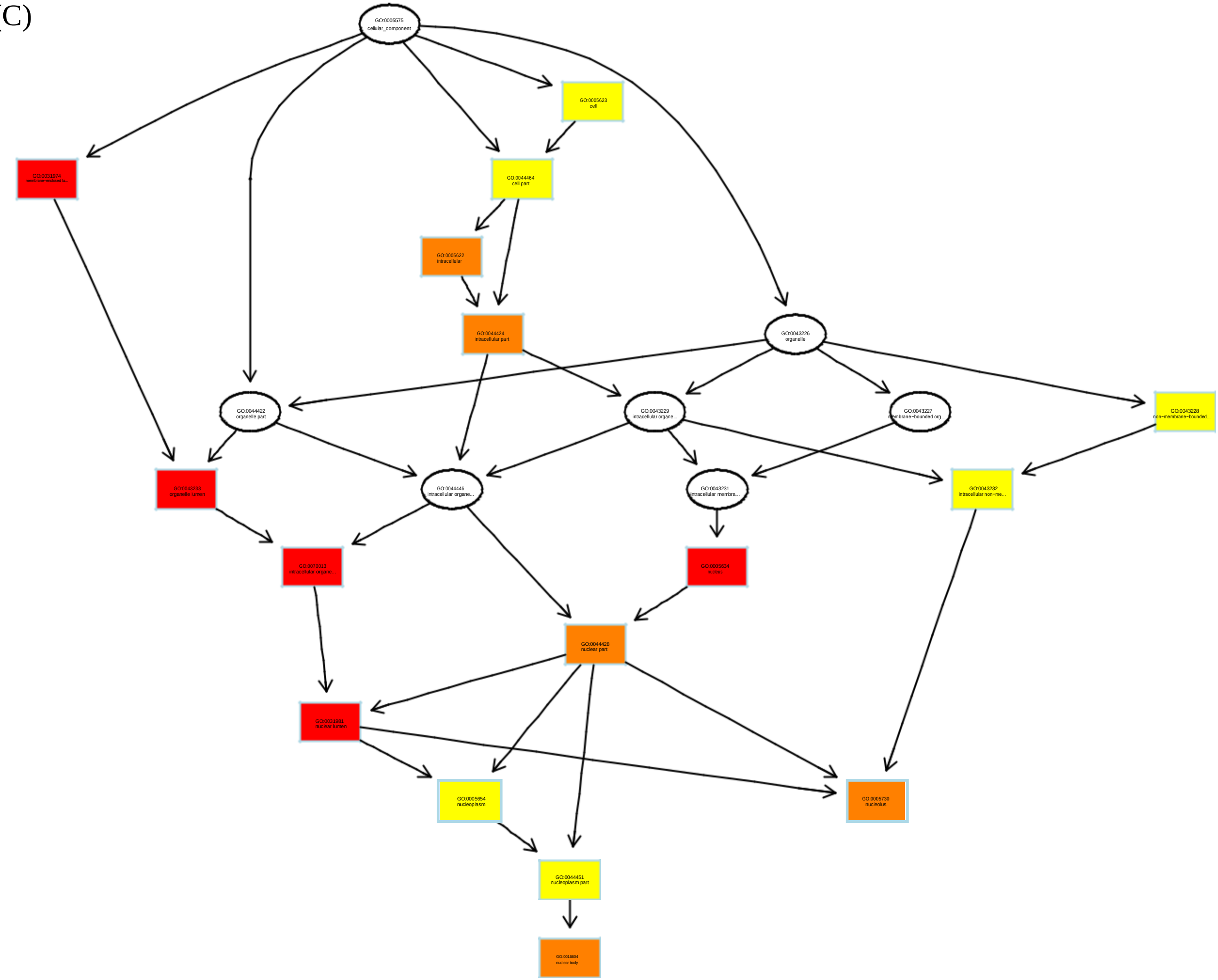
GO:0005575
cellular_component
GO:0005623
cell
GO:0031974
membrane−enclosed lu...
GO:0044464
cell part
GO:0005622
intracellular
GO:0044424
intracellular part
GO:0043226
organelle
GO:0044422
organelle part
GO:0043229
intracellular organe...
GO:0043227
membrane−bounded org...
GO:0043228
non−membrane−bounded...
GO:0043233
organelle lumen
GO:0044446
intracellular organe...
GO:0043231
intracellular membra...
GO:0043232
intracellular non−me...
GO:0070013
intracellular organe...
GO:0005634
nucleus
GO:0044428
nuclear part
GO:0031981
nuclear lumen
GO:0005654
nucleoplasm
GO:0005730
nucleolus
GO:0044451
nucleoplasm part
GO:0016604
nuclear body

S1 Fig: GO enrichment analysis of the predicted RNAi genes **(A)** biological process, **(B)** molecular function and **(C)** cellular process. In the directed acyclic graph (DAG) the downstream term corresponds to a subset of the upstream term. The significant ($P < 0.05$, $FDR < 0.05$) GO terms are in colored boxes (the degree of color saturation is positively correlated to the enrichment level of the GO term), and non-significant terms are in white boxes.
