# Supplementary material for: In silico identification and characterization of AGO, DCL and RDR gene families and their associated regulatory elements in sweet orange (Citrus sinensis L.)
Source: PLoS One. 2020 Dec 21;15(12):e0228233. doi: 10.1371/journal.pone.0228233 (PMC7751981; doi:10.1371/journal.pone.0228233)
Supplement: S2 Fig — Rows of the figure represent the predicted RNAi genes and the columns represent the families of the TFs. The number indicates the TF families regulate the RNAi genes. (PDF) [file pone.0228233.s012.pdf]

	AP2	ARF	B3	BBR-BPC	BES1	bHLH	bZIP	C2H2	CAMTA	Dof	EIL	ERF	GATA	GRAS	HD-ZIP	LBD	MIKC_MADS	MYB	NAC	Nin-like	SBP	TALE	TCP	Trihelix	WOX	WRKY	ZF-HD
CsDCL1	1		1		2		3	1	1	4			1			1	1		1							20	
CsDCL2											2								5								1
CsDCL3	1			2	3	1	2			4				1			1										
CsDCL4				1								5							3		1	1	6	1		4	
CsAGO1					1	5	10												17								
CsAGO4	1																				1						
CsAGO5a	1			2						6		28	1	1		4	1	2				1					
CsAGO5b								1				2													1		
CsAGO5c								1				10				3	1			1							
CsAGO6								1						1	1												
CsAGO7	1			1				1		3		1					1	2	7								
CsAGO10	1		1	2				1		4			3				1	2									
CsRDR1							2																				
CsRDR2												2											5			3	
CsRDR3		1	1														1										
CsRDR6												1															

S2 Fig: Distribution of TF families corresponding to genes. Rows of the figure represent the predicted RNAi genes and the columns represent the families of the TFs. The number indicates the TF families regulate the RNAi genes.
